# Human Leukocyte Antigen (HLA)-DRB1*15:01 and HLA-DRB5*01:01 Present Complementary Peptide Repertoires

**DOI:** 10.3389/fimmu.2017.00984

**Published:** 2017-08-21

**Authors:** Erika Margaret Scholz, Miguel Marcilla, Xavier Daura, David Arribas-Layton, Eddie A. James, Iñaki Alvarez

**Affiliations:** ^1^Institut de Biotecnologia i de Biomedicina, Universitat Autònoma de Barcelona, Bellaterra, Spain; ^2^Immunology Unit, Department of Cell Biology, Physiology and Immunology, Universitat Autònoma de Barcelona, Bellaterra, Spain; ^3^Proteomics Unit, Centro Nacional de Biotecnología (CSIC), Madrid, Spain; ^4^Catalan Institution for Research and Advanced Studies (ICREA), Barcelona, Spain; ^5^Benaroya Research Institute at Virginia Mason, Seattle, WA, United States

**Keywords:** human leukocyte antigen-DR, binding motif, mass spectrometry, peptidome, multiple sclerosis

## Abstract

Human leukocyte antigen (HLA)-DR15 is a haplotype associated with multiple sclerosis. It contains the two DRB* genes DRB1*1501 (DR2b) and DRB5*0101 (DR2a). The reported anchor motif of the corresponding HLA-DR molecules was determined in 1994 based on a small number of peptide ligands and binding assays. DR2a could display a set of peptides complementary to that presented by DR2b or, alternatively, a similar peptide repertoire but recognized in a different manner by T cells. It is known that DR2a and DR2b share some peptide ligands, although the degree of similarity of their associated peptidomes remains unclear. In addition, the contribution of each molecule to the global peptide repertoire presented by the HLA-DR15 haplotype has not been evaluated. We used mass spectrometry to analyze the peptide pools bound to DR2a and DR2b, identifying 169 and 555 unique peptide ligands of DR2a and DR2b, respectively. The analysis of these sets of peptides allowed the refinement of the corresponding binding motifs revealing novel anchor residues that had been overlooked in previous analyses. Moreover, the number of shared ligands between both molecules was low, indicating that DR2a and DR2b present complementary peptide repertoires to T cells. Finally, our analysis suggests that, quantitatively, both molecules contribute to the peptide repertoire presented by cells expressing the HLA-DR15 haplotype.

## Introduction

Human leukocyte antigen (HLA)-DP, -DQ, and -DR are membrane heterodimeric glycoproteins, composed by two chains, α and β, encoded in the class II region of the human major histocompatibility complex (MHC) or HLA. Thus, four different HLA-DP and HLA-DQ molecules are present in heterozygous cells, as each polymorphic α chain can interact with either polymorphic β chain. In the case of HLA-DR, the gene encoding the HLA-DRα subunit is dimorphic (and both forms are functionally equivalent). As such, only two different heterodimers are usually expressed. However, some haplotypes include two functional *HLA-DRB* genes, allowing the expression of four different molecules in heterozygous individuals, all of them carrying the same HLA-DRα chain. Specifically, the DR3, DR11, DR12, DR13, and DR14 haplotypes express *DRB1* and *DRB3*; DR4, DR7, and DR9 express *DRB1* and *DRB4*; and DR15 and DR16 express *DRB1* and *DRB5*. The fact that some HLA haplotypes carry two different *HLA-DRB* genes suggests that this provide some advantages regarding the presentation of peptides derived from pathogens to CD4^+^ T cells.

It has been known for a long time that different HLA-DR molecules can present common peptides, indicating that HLA-DR molecules are to a certain level promiscuous. In fact, in the HLA class II, antigen processing pathway CLIP, derived from the invariant chain (Ii), must bind essentially all HLA-II molecules for a correct peptide selection. In addition, many other promiscuous binders have been described so far (1–11).

Nevertheless, although the promiscuity of HLA-DR molecules is accepted, the degree of overlap between different peptide repertoires has not been extensively addressed. In this regard, we compared the peptide repertoires of four HLA-DR allotypes differentially associated with rheumatoid arthritis and found a low degree of promiscuity in their bound peptidomes (12).

Human leukocyte antigen-DR15, a haplotype that expresses two functional HLA-DRB genes, is associated with multiple sclerosis (MS). MS is a chronic inflammatory disease of the central nervous system and is considered a T cell-mediated autoimmune disorder with a prevalence of 0.5–1.5 per 1,000 inhabitants in the northern hemisphere [reviewed in Ref. (13)]. The pathology is characterized by inflammation, demyelination, and axonal degeneration (14), and, as other autoimmune disease, its etiology is not simple, involving multiple genetic and environmental factors. As said above, the HLA-DR15 haplotype is the strongest single genetic factor associated with MS with a reported odds ratio of 3.08 (15). This haplotype expresses *DRB1* and *DRB5* genes, among which HLA-DRB1*15:01 (DR2b) and HLA-DRB5*01:01 (DR2a) are the most prevalent alleles. The anchor motifs for DR2a and DR2b were described by several groups two decades ago (16, 17) based on a few peptides sequenced by Edman degradation and binding assays. Recently, a higher number of ligands of these allotypes have been described deriving from a low number of parental proteins, principally HLA-II and others proteins of the endogenous pathway (18). In addition, the Immuno Epitope Database and Analysis Resource (19) (http://www.iedb.org/) reports binding data for several hundreds of peptides in relation to different HLA-DR allotypes, including DR2a and DR2b. However, the limited number of peptide eluted from these allotypes, which are the most relevant peptides presented *in vivo*, and peptides evaluated in binding assays means that the reported motifs may actually not include all potential anchor residues.

The co-expression of both DR2a and DR2b at the RNA level and on the cell surface has been demonstrated in different cell types (20). In that study, RNA levels were shown to be higher for DR2a, although protein expression on the cell surface seemed to be similar for both allotypes. Nevertheless, the contribution of the peptides presented by both HLA-DR molecules to the peptide repertoire presented by this haplotype and the degree of overlap between them remains unclear.

In this study, we have addressed three main questions: first, the fine-mapping of the anchor motifs of DR2a and DR2b by the identification of natural ligands bound to these allotypes; second, the degree of overlap of the peptide repertoires bound to these two HLA-DR molecules (DR2a and DR2b); and third, the contribution of DR2a and DR2b to the peptide repertoire presented on the cell surface. To that end, we used liquid chromatography couple to tandem mass spectrometry (LC–MS/MS) to characterize the peptide repertoires associated with HLA-DRB1*15:01 (555 unique peptides) and HLA-DRB5*01:01 (169 unique peptides) from bare lymphocyte syndrome (BLS) transfected cells. This analysis allowed us to refine the binding motifs of these allotypes, identifying some novel anchor residues. The analysis of these peptide pools indicated that, although these molecules can share some peptides ligands, the overlap of both peptidomes was extremely low. Finally, our estimations indicate that both allotypes contribute similarly to the peptide repertoire presented by HLA-DR15 to CD4^+^ T cells.

## Materials and Methods

### Cell Lines and Antibodies

The BLS patient-derived B cell line (BLS) transfected with the genes encoding the molecules DRB1*15:01 (BLS-DR2b) or DRB5*01:01 (BLS-DR2a) were kindly provided by G. Nepom and W. Kwok (University of Washington, Seattle, WA, USA) and were used as the source of the HLA-DR peptide ligands.

For the purification of the peptide pools associated with DRB1*15:01 or DRB5*01:01 the monoclonal antibody B8.11.2 (IgG2b) was used. This antibody recognizes a DR framework structure present on all types of HLA-DR molecules (21, 22).

### Purification of the HLA-DR-Associated Peptide Pools

Peptides were purified as previously described (23, 24). About 1 × 10^9^ cells were lysed in tris buffer (TB: 50 mM Tris/HCl (pH 7.6), 150 mM NaCl) with 1% Igepal-CA630 supplemented with protease inhibitors (cOmplete Protease Inhibitor Cocktail Tablet, Hoffman-La Roche, Basel, Switzerland) at 4°C for 2 h. Lysates were centrifuged for 10 min at 1,300 × *g*, and supernatants were ultra-centrifuged for 1 h at 100,000 × *g*. The soluble fraction was precleared using a Tris-blocked sepharose column (CNBr-activated sepharose, GE Healthcare Bio-Sciences AB, Sweden). The flow-through was incubated overnight at 4°C with B8.11.2-sepharose and washed as follows: first, with 50 ml of TB, 0.5% Igepal-CA630; then, with 200 ml of TB; and, finally, with 500 ml of Tris–HCl pH 7.6, 5 mM NaCl. HLA-DR–peptide complexes were eluted in 0.1% trifluoroacetic acid. Protein-containing fractions were determined by the Bradford method, pooled and concentrated in a SpeedVac (Thermo Fisher scientific, Waltham, MA, USA). Peptides were purified by ultra-filtration in a Centricon-10 device (Millipore, Ireland, Ltd.). Retained material, containing HLA-DRα and β chains are shown in Figure S1 in Supplementary Material.

### LC–MS/MS Analysis

Samples were analyzed in a nano-LC ultra HPLC (Eksigent) coupled online with a 5,600 triple TOF mass spectrometer (AB Sciex) and equipped with a C18 chromXP trapping column (350 µm × 0.5 mm, 3 µm, Eksigent) and a C18 chromXP column (75 µm × 150 mm, 3 µm, Eksigent). Solvent A and B were 0.1% formic acid and 0.1% formic acid in acetonitrile, respectively. Peptides were fractionated at a flow-rate of 300 nl/min at 40°C under gradient elution conditions, as follows: isocratic conditions of 2% B for 1 min, a linear increase to 30% B in 181 min, a linear increase to 40% B in 23 min, a linear increase to 90% B in 15 min, 90% B for 10 min, and back to initial conditions. Total runtime was 250 min. For blank injections, a shorter gradient was employed consisting of isocratic conditions of 5% B for 1 min, a linear increase to 30% B in 109 min, a linear increase to 40% B in 10 min, a linear increase to 90% B in 5 min, 90% B for 5 min, and back to initial conditions. In this latter case, total runtime was 150 min. Each acquisition cycle comprised a survey scan (350–1,250 *m/z*) of 250 ms and up to of 25 MS/MS scans (100–1,500 *m/z*) of 100 ms. The HPLC and the mass spectrometer were, respectively, controlled with Eksigent Control (version 3.12, Eksigent) and Analyst TF (version 1.7, Eksigent). Total ion chromatogram of a blank and the samples and fragmentation spectra of two peptides are shown in Figure S2 in Supplementary Material. The MS/MS data have been deposited to the ProteomeXchange Consortium *via* the PRIDE partner repository (25) with the dataset identifier PXD006534.

### MS/MS Ion Search and Peptide Identification

Raw MS/MS data were converted to mgf files with Peakview 1.2 (AB SCiex) and searched against a concatenated target-decoy database containing the 88,669 Uniprot entries of the Homo sapiens complete proteome set (as of March 2015) and their corresponding reverse sequences. The mgf file corresponding to the DR2b fraction was recalibrated with Protein Pilot (version 4.5, AB Sciex) before the search. MASCOT (Matrix Science, version 2.5) was used as search engine with the following parameters: no enzyme, MS tolerance of 15 ppm, MS/MS tolerance of 0.025 Da and protein N-terminal acetylation, pyroglutamic acid formation from glutamine, and methionine oxidation as variable modifications. Estimation of the false discovery rate (FDR) was carried out by decoy hit counting as previously described (26). Results were filtered at a FDR <5% at the peptide level.

### Identification of the Peptide-Binding Core

All the peptides associated with DR2a or DR2b were analyzed with NetMHCIIpan 3.1 Software (27) to predict the binding core of each peptide. After the elimination of the redundant cores in nested sets, the remaining 9-mer cores were analyzed as previously described (28, 29). Preferences in residue usage were determined by comparing the observed frequency of each amino acid at each peptide position (fobs) with the frequency of the same amino acid in the database (fexp), named as deviation from mean in proteome (DMP) using a binomial test with Bonferroni’s correction. *P*-values <0.05 were considered statistically significant. Residues with a statistically significant increase after Bonferroni correction and a DMP value >2.5 were considered to be the main anchor residues. Secondary residues were those with a DMP value >1.5.

### Modeling of the Peptide–HLA-DR Interaction

Modeling of complexes between HLA-DR2a and HLA-DR2b and the different peptides was performed using a simulation protocol detailed elsewhere (12, 30). The binding score delivered by this procedure can be used to estimate the relative binding affinity of different peptides for the HLA molecules.

### Binding Assays

The experimental affinity of different peptides to HLA-DR molecules was determined as previously described (31). Briefly, recombinant DR2b (DRA1*01:01/DRB1*15:01) and DR2a (DRA1*01:01/DRB5*01:01) proteins were used for peptide-binding assays. Increasing concentrations of each non-biotinylated test peptide were incubated in competition with 1 µM of the biotinylated GAD555-567 peptide (NLIRVVSSNRAAT, modified from NFFRMVISNPAAT to bind to DRB1*15:01 and DRB5*01:01 in a single unambiguous register) in wells coated with HLA-DR2a or HLA-DR2b. After washing, residual biotinylated reference peptide was labeled using europium conjugated streptavidin (Perkin Elmer) and quantified using a Victor3 D time resolved fluorometer (Perkin Elmer). Peptide-binding curves were simulated by non-linear regression with Prism Software (version 4.03, GraphPad Software Inc.) using a sigmoidal dose–response curve. IC50 binding values were calculated from the resulting curves as the peptide concentration needed for 50% inhibition of reference peptide binding. Equivalent amounts of purified HLA-DR protein were plated on each plate, but similar amounts of total protein can have different levels of activity. Thus, different preparations of protein frequently give different levels of maximum counts (presumably based on differences in purity and the proportion of the protein that is functional). Indeed differences between different preparations of the same HLA-DR protein can be observed. However, the observed IC50 values do not vary based on the maximum number of counts, probably because the labeled and unlabeled peptides are still competing for an equal number of binding sites.

## Results

### Characterization of the Peptide Repertoires Bound to DR2a and DR2b Molecules

BLS-DR2a and BLS-DR2b cells were used as the source of the peptide–HLA-DR complexes. After purification by immunoaffinity chromatography, the trimeric complexes were denatured under acidic conditions and their associated peptides purified by ultra-filtration. The resulting peptide mixtures were analyzed by LC–MS/MS and peptide identification was carried out using Mascot as search engine. Peptides with a length of 11 amino acids or longer were considered for the analysis. A total of 177 and 560 peptide ligands were identified from the DR2a and DR2b molecules, respectively. Peptides derived from some heterogeneous nuclear ribonucleoproteins were considered as background contaminants on the basis of their particular features (their sequences were notably rich in Gly and Pro) and of our previous observations [similar peptides had been found in previous analysis of other unrelated HLA-II and HLA-I molecules (32)]. Thus, a final list of 169 unique peptides for DR2a and 555 unique peptides for DR2b were considered. The lists of these peptides and the corresponding parental proteins are shown in Tables S1 and S2 in Supplementary Material.

**Figure 1:**
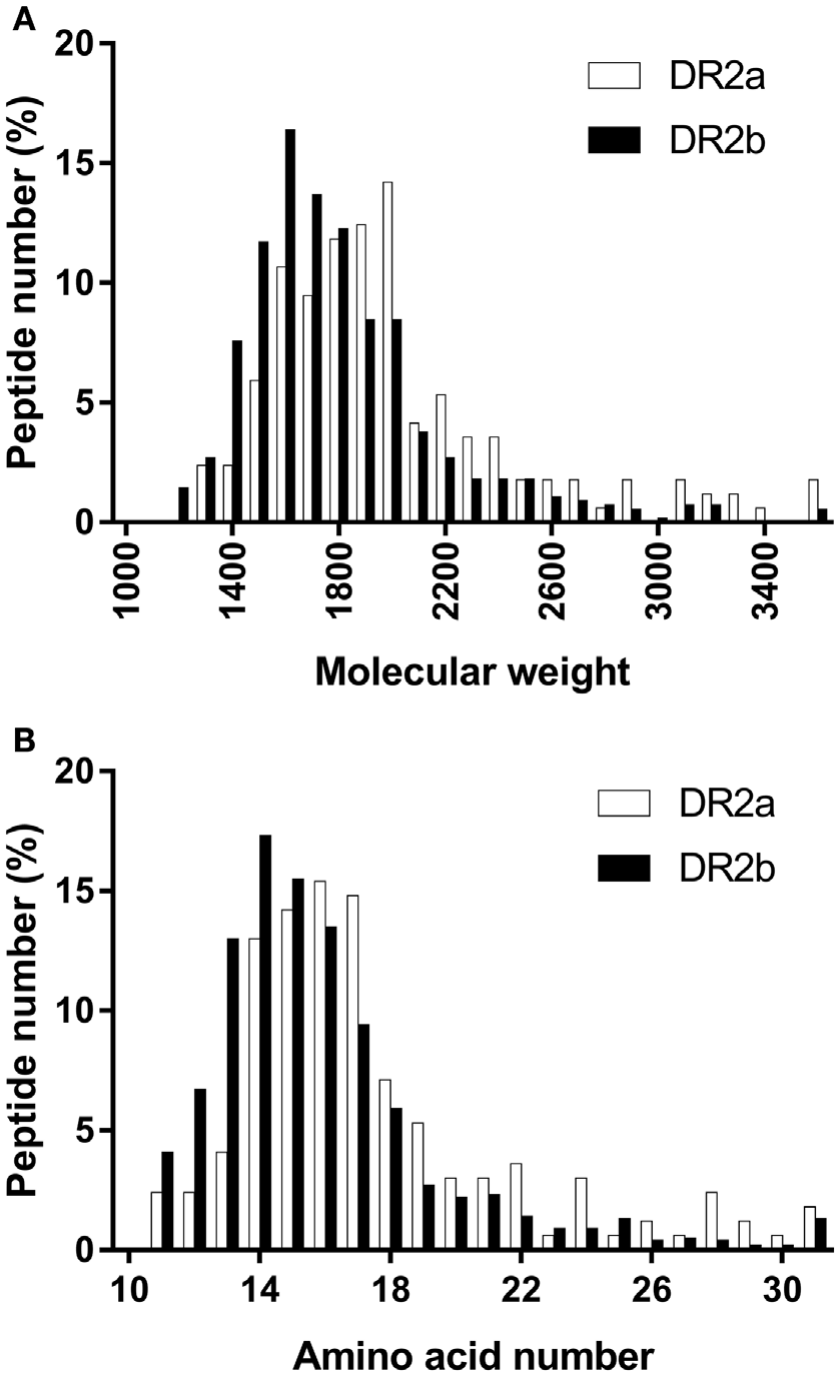
Peptide size distribution of the peptides sequenced from the different human leukocyte antigen-DR allotypes. **(A)** Groups of 100 Da were selected. Values shown are the percentage of peptides in each group. **(B)** Values shown are the percentage of peptides with different amino acid number.

The size distribution of the peptide ligands sequenced from the DR2 allotypes followed a normal distribution with an average molecular weight of 1,977.9 and 1,761.0 Da (Figure 1A) and a length of 17.6 and 15.9 residues for DR2a and DR2b, respectively (Figure 1B).

**Figure 2:**
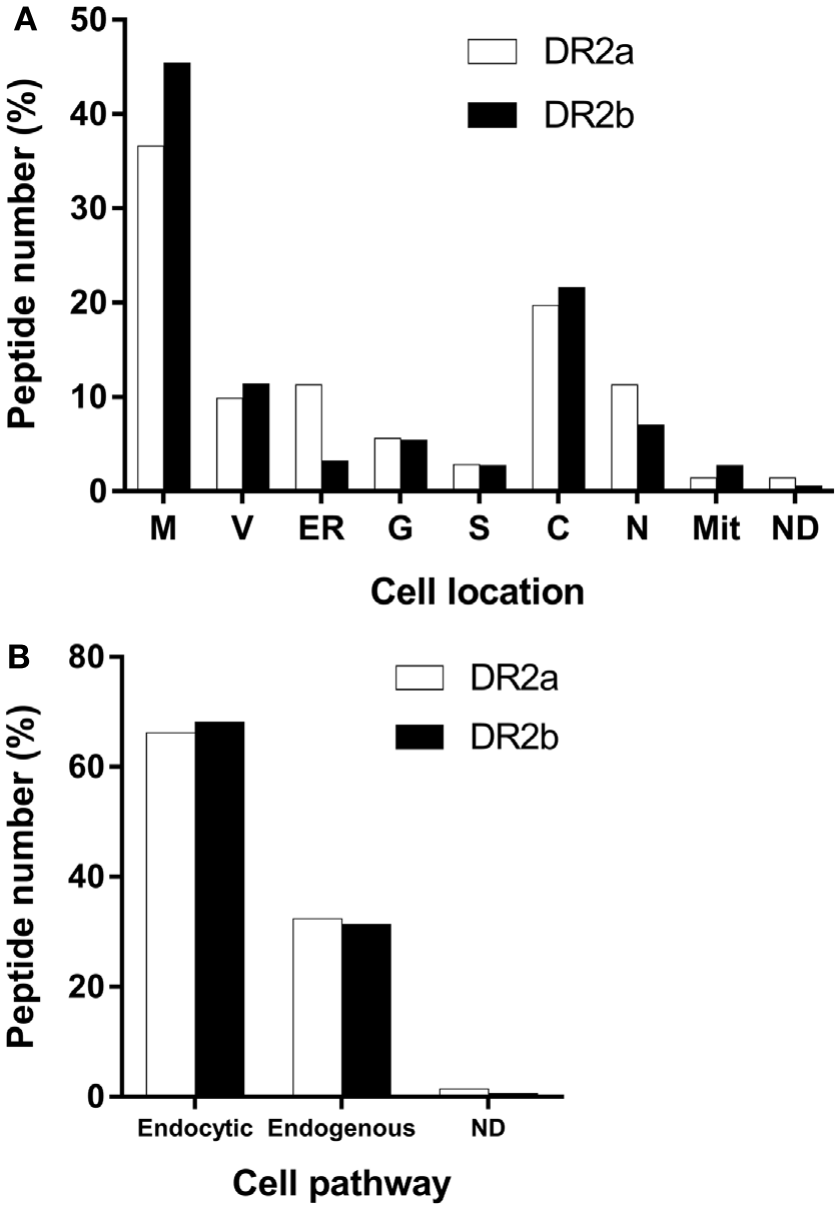
Major cell location of the parental proteins of the peptides sequenced from the different human leukocyte antigen-DR molecules. **(A)** Values shown are the percentage of proteins for each cellular location. M, membrane; V, vesicular compartments; ER, endoplasmic reticulum; G, Golgi apparatus; S, secreted; C, cytosol; N, nucleus; Mit, mitochondria; ND, not determined. **(B)** Values shown are the percentage of proteins in each cellular pathway. Endocytic pathway comprises M, V, ER, G, and S. Endogenous pathway includes C, N, and M.

Most of the HLA-II ligands were peptides derived from proteins located in the endocytic pathway, although some peptides came from cytosolic or nuclear proteins. To test if the DR2a- and DR2b-derived peptidomes were canonical regarding the subcellular location of their source proteins, we analyzed this parameter and found that most of them were located in vesicular compartments (Figure 2A). The fraction of parental proteins of the HLA-DR ligands located in the endocytic pathway was similar for DR2a (66.2%) and DR2b (68.1%) (Figure 2B). These observations confirmed that most of the peptides identified correspond to bona fide HLA-DR ligands.

### Refinement of the HLA-DRB1*15:01 and HLA-DRB5*01:01 Binding Motifs

**Figure 3:**
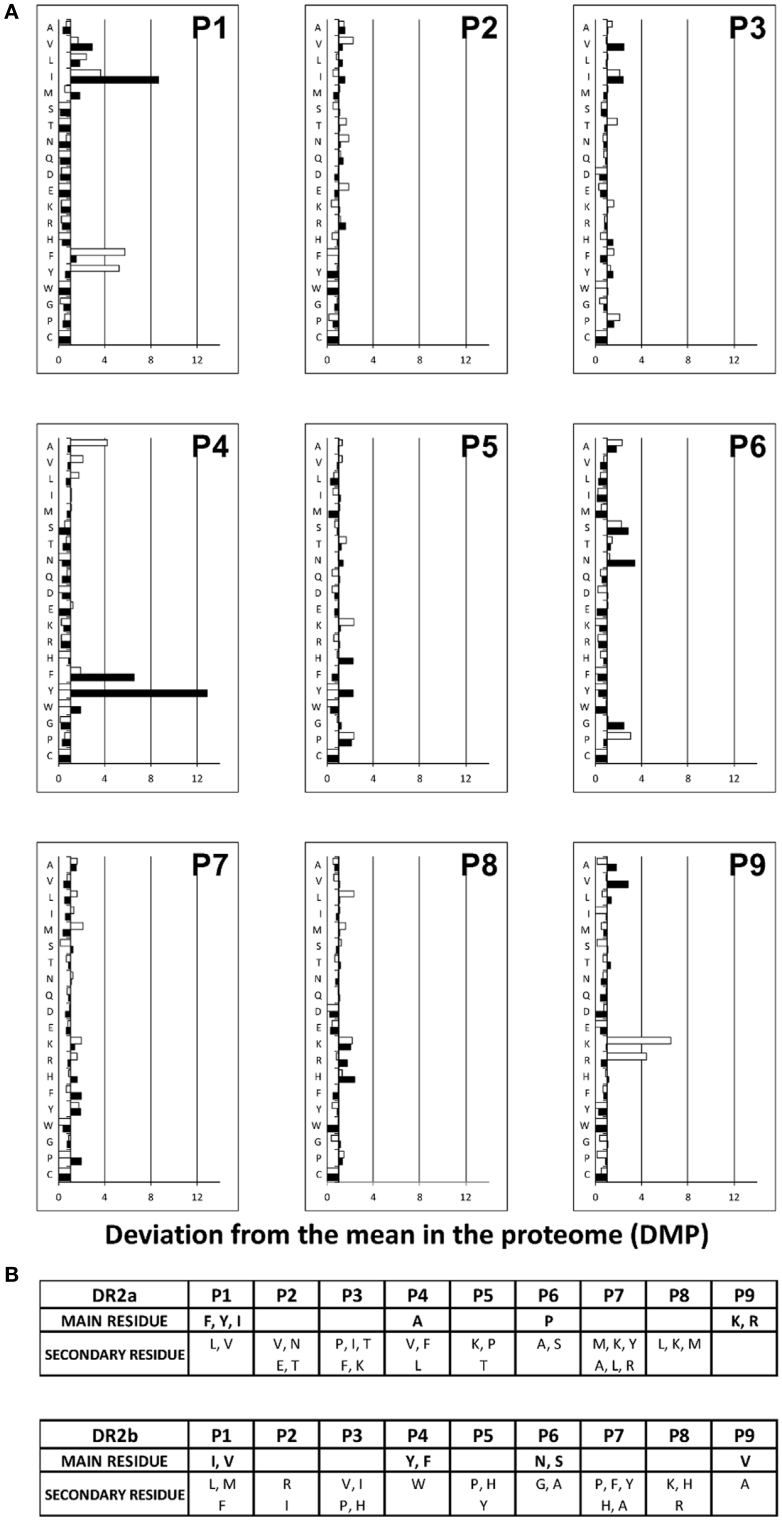
Refined anchor motifs for DR2a and DR2b molecules. **(A)** Residue use among DR2a (white) and DR2b (black) natural ligands. For each of the peptide core positions (P), the percent frequency of each amino acid was calculated. Normalized values were calculated as the number of times that each relative frequency was increased or decreased relative to the mean frequency of the corresponding residue among human proteins (deviation from the mean in the proteome [DMP]). DMP values are presented as histograms. **(B)** DR2a and DR2b peptide-binding motifs. Residues with a statistically significant increase after Bonferroni correction (top) and a DMP value >2.5 were considered to be the main anchor residues. Secondary residues are also shown, which were those with a DMP value >1.5.

The HLA-DRB1*15:01 and HLA-DRB5*01:01 binding motifs were described years ago (16, 17). The described motif for binding to DR2a included bulky hydrophobic residues at the P1 core position, Val, Ile, Met, or Gln at P4 and positively charged residues at P8 or P9. For DR2b, aliphatic residues (Leu, Val, or Ile) were preferred to Phe or Tyr at P1 and Met, Ile, Val, or Phe were favored at P4 and P7. Importantly, these anchor motifs were based only on the sequences of a limited number of peptides eluted from these allotypes and a few binding assays with selected peptides. It would not be unlikely that some anchor residues were not detected as part of the binding motif. Thus, in order to more robustly characterize the binding preferences of DR2a and DR2b, we identified the theoretical cores and calculated the binding affinity with NetMHCIIpan 3.1 Server (27). To this end, each peptide was assayed against its corresponding HLA-DR allotype. If the same binding core was obtained for different peptides belonging to a nested set, this was considered only once in the analysis. As a result, 89 unique cores for DR2a and 270 for DR2b were considered. As shown in Figure 3, the anchor motifs described for each allotype were roughly similar to those reported previously. However, some differences could be observed. Thus, in the case of DR2a, the main anchor positions were P1, P4, P6, and P9. At P1, the preferred residues were Phe, Tyr, and Ile, and at P4 the only amino acid significantly overrepresented after the Bonferroni’s correction was Ala, which was not included in the previously defined motif. At P6, Pro, Ala, and Ser were favored. Finally, in P9, Lys and Arg were clearly the most frequent residues. In DR2b, P1, P4, P6, and P9 were also the main anchor residues. In P1, Ile and Val were the most favored amino acids. In P4, large aromatic residues Tyr and Phe were selected, while in P6 the polar amino acids Asn and Ser were preferred. Finally, the frequencies of Val were increased in P9 (Figure 3).

### Low Similarity of the Peptide Repertoires Associated with DR2a and DR2b

Since the peptide-binding motifs of DR2a and DR2b showed some common features, we set out to analyze the degree of overlap of the two peptide pools. In our dataset, only 13 peptides were identified from both allotypes (7.7% for DR2a and 2.3% for DR2b) (Table 1). It is worth noting that both allotypes, and specially DR2a, displayed a large number of peptides derived from Ii, including six shared peptides derived from Ii. If these ligands were not included in the analysis, only seven peptides (4.1% for DR2a and 1.3% for DR2b) would be shared by both HLA-DR molecules.

**Table 1:** Shared peptides between DR2a and DR2b.

#	Protein	Peptide
1	Cofilin-1	ASGVAVSDGVIKVFNDMKVR
2	Cofilin-1	ASGVAVSDGVIKVFNDMKVRK
3	Integrin beta	NIQPIFAVTSRMVKTYE
4	Invariant chain	KPPKPVSKMRMATPLLMQA
5	Invariant chain	KPPKPVSKMRMATPLLMQALP
6	Invariant chain	LPKPPKPVSKM
7	Invariant chain	LPKPPKPVSKMRMATPLLMQAL
8	Invariant chain	LPKPPKPVSKMRMATPLLMQALP
9	Invariant chain	LPKPPKPVSKMRMATPLLMQALPM
10	Nuclease-sensitive element-binding protein 1	PPAENSSAPEAEQGGAE
11	Protein CutA	PALLPVASRLLLLP
12	SWI/SNF complex subunit SMARCC2	PGTPLPPDPTAPSPGTVTPVPPPQ
13	SWI/SNF complex subunit SMARCC2	PTAPSPGTVTPVPPPQ

We recently sequenced about 200 HLA-II-bound peptides from the DR2-homozygous B cell lymphoblastoid cell line (B-LCL) MGAR (12). Among those, 22 peptides, grouped in 15 nested sets, were also identified in this work, including 8 peptides (6 nested sets) associated with DR2a and 14 (9 nested sets) with DR2b (Table 2). Remarkably, not a single peptide was found in the three repertoires confirming the low degree of overlap in the peptide repertoires associated with DR2a and DR2b.

**Table 2:** Common peptides from MGAR and BLS-DR2a or BLS-DR2b.

PEPTIDE	SEQUENCED FROM			NetMHCIIPan 3.1				Matrix of this paper				BINDING [IC50 (nM)]		MODELING (ΔG, kJ/mol)		
				
	DR2a	DR2b	MGAR	CORE DR2a	IC 50 (nM) DR2a	CORE DR2b	IC 50 (nM) DR2b	Core DR2a	Score DR2a	Core DR2b	Score DR2b	DR2a	DR2b	CORE^a^	DR2a	DR2b
LEEFGRFASFEAQG		X	X	FGRFASFEA	**275.33**	FGRFASFEA	500.20	FGRFASFEA	13.411	FGRFASFEA	**16.571**	>50 μM	>50 μM	FGRFASFEA	−41	**−55**
LEEFGRFASFEAQGA		X	X													
ADDGKIVIFQSKPEIQ		X	X	IVIFQSKPE	564.48	IVIFQSKPE	**213.78**	IVIFQSKPE	15.037	IVIFQSKPE	**24.163**	ND	ND	IVIFQSKPE	−58	**−150**
APGAGSLALFPGIRLE		X	X	LALFPGIRL	148.05	LALFPGIRL	**112.11**	SLALFPGIR	13.674	LALFPGIRL	**18.801**	ND	ND	LALFPGIRL	−50	**−97**
LPSEKAIFLFVDKTVPQ		X	X	FLFVDKTVP	1,108.20	IFLFVDKTV	**362.52**	KAIFLFVDK	12.652	IFLFVDKTV	**22.154**	–	14.5 μM	IFLFVDKTV	−52	**−86**
LPSEKAIFLFVDKTVPQS		X	X													
LVRVVVPYQGPSSDY		X	X	VRVVVPYQG	1,365.31	VRVVVPYQG	**460.01**	VRVVVPYQG	13.268	VVPYQGPSS	**22.408**	ND	ND	VRVVVPYQG	−35	**−100**
														VVPYQGPSS	−80	**−114**
NEQKLNRYPASSLVVVR		X	X	LNRYPASSL	181.61	LNRYPASSL	**150.87**	RYPASSLVV	13.245	LNRYPASSL	**20.546**	ND	ND	LNRYPASSL	−82	**−94**
QKKEIHLYQTFVVQ		X	X	IHLYQTFVV	577.16	IHLYQTFVV	**86.23**	IHLYQTFVV	9.231	IHLYQTFVV	**26.846**	>50 μM	>50 μM	IHLYQTFVV	−42	**−102**
QKKEIHLYQTFVVQL		X	X						9.231							
QKKEIHLYQTFVVQLQDPR		X	X					QTFVVQLQD	10.604							
QKKEIHLYQTFVVQLQDPREP		X	X						10.604							
QPGVLIQVYEGERAM		X	X	IQVYEGERA	660.48	IQVYEGERA	**487.63**	LIQVYEGER	12.470	IQVYEGERA	**27.822**	ND	ND	IQVYEGERA	−61	**−123**
TPKIQVYSRHP		X	X	PKIQVYSRH	**1,423.97**	PKIQVYSRH	3,407.84	TPKIQVYSR	11.077	KIQVYSRHP	**13.167**	ND	ND	PKIQVYSRH	−77	**−100**
ADIQTERAYQKQP	X		X	IQTERAYQK	**190.72**	IQTERAYQK	3,028.33	IQTERAYQK	**19.227**	IQTERAYQK	17.163	ND	ND	IQTERAYQK	−58	**−117**
ELEELRAEQQRLKSQD	X		X	LRAEQQRLK	**140.55**	LRAEQQRLK	2,413.08	LRAEQQRLK	**17.190**	LRAEQQRLK	9.480	2.4 μM	>50 μM	LRAEQQRLK	**−116**	−107
ELEELRAEQQRLKSQDL	X		X							QQRLKSQDL	10.511					
NPPDIVVQPGHIR	X		X	IVVQPGHIR	**131.27**	IVVQPGHIR	1,690.71	IVVQPGHIR	16.488	IVVQPGHIR	**19.347**	ND	ND	IVVQPGHIR	−47	**−76**
QTKEFQVLKSLGKLA	X		X	FQVLKSLGK	**5.89**	FQVLKSLGK	62.71	FQVLKSLGK	**21.571**	FQVLKSLGK	13.238	0.08 μM	>50 μM	FQVLKSLGK	**−88**	−56
QTKEFQVLKSLGKLAMG	X		X													
SQAEFEKAAEEVRHL	X		X	FEKAAEEVR	**493.62**	FEKAAEEVR	5,881.78	FEKAAEEVR	**20.382**	QAEFEKAAE	10.940	5.8 μM	>50 μM	FEKAAEEVR	**−112**	−82
VAIVQAVSAHRHR	X		X	IVQAVSAHR	**4.87**	IVQAVSAHR	35.95	IVQAVSAHR	**20.445**	IVQAVSAHR	18.858	ND	ND	IVQAVSAHR	**−154**	−138

*^a^For the peptide LVRVVVPYQGPSSDY, the two cores obtained with NetMHCIIPan 3.1 and the score of this work are shown*.

*ND, not determined*.

*Bold indicates the DR molecule with the best score*.

### Contribution of HLA-DRB1*15:01 and HLA-DRB5*01:01 to the Repertoire of HLA-DR15

To analyze the contribution of the peptides eluted from DR2a and DR2b to the global peptidome presented by the haplotype DR15, we analyzed the peptide pool sequenced in the present work and in a previous report from the B-LCL, MGAR (12). First, we used the NetMHCIIpan 3.1 Software to identify the putative MHC-interacting peptide cores. This program computes a theoretical binding affinity for each peptide. Using this approach, 88.8% of the peptides identified from BLS-DR2a cells presented higher affinity for DR2a than for DR2b (Table S3 in Supplementary Material). By contrast, only 43.4% of the natural DR2b ligands showed higher affinity for DR2b than for DR2a (Table S4 in Supplementary Material). In an attempt to tackle this apparent contradiction, we carried out a second approach using the refined binding motif described in the present work. A new score was generated for all the 9-mer cores by adding up the DMPs values obtained of each residue at each position. With this new scoring scheme, 85.8% of the peptides sequenced from DR2a had a higher affinity for DR2a than for DR2b (Table S5 in Supplementary Material). Most importantly, the fraction of peptides with a higher affinity for DR2b in the peptide pool obtained from this allotype increased from 43.4 to 86.1% (Table S6 in Supplementary Material). These data indicate that the empirical information obtained from the characterization of MHC-bound peptidomes is complementary to that provided by bioinformatics tools such as NetMHC2pan 3.1. Moreover, as shown here, it can improve the prediction of binding affinities and contribute to define more accurate anchor motifs.

This same approach was conducted with the peptide pool isolated from the MGAR cell line. Using that dataset and the here defined score, we estimate that 51.1% of the peptides displayed by DR15 are presented by DR2a and the remaining 48.9% by DR2b (Table S7 in Supplementary Material). Therefore, our data suggest that, in the DR15 haplotype, the contribution of both HLA-DR molecules to the conformation of the HLA-DR15 peptide repertoire is similar, at least in quantitative terms.

### Binding Assay to HLA-DR Molecules

To gain insight into the molecular features that govern the interaction of DR15 with its peptide ligands we considered the 8 and 14 peptides associated, respectively, with DR2a and DR2b that we described in a previous report (12) (Table 2).

We calculated their theoretical binding affinities with NetMHCIIpan 3.1. All the sequences tested showed a high affinity (IC50 < 1,000 nM) except the peptide TPKIQVYSRHP, derived from β2-microglobulin, which probably is not a real ligand, but a contaminant. Of note, all the peptides presented higher affinity for the allotype from which they were sequenced, with the exceptions of the nested set LEEFGRFASFEAQG(A) and the peptide TPKIQVYSRHP, which were identified from the BLS-DR2b cell line but showed a higher theoretical affinity for DR2a (Table 2). Interestingly, when the theoretical affinity was computed with the empirical scoring scheme generated in the present work, all the sequences presented a higher affinity for the HLA-DR molecule from which they derived except the peptide NPPDIVVQPGHIR, sequenced from DR2a and with a theoretical higher affinity for DR2b (Table 2).

**Figure 4:**
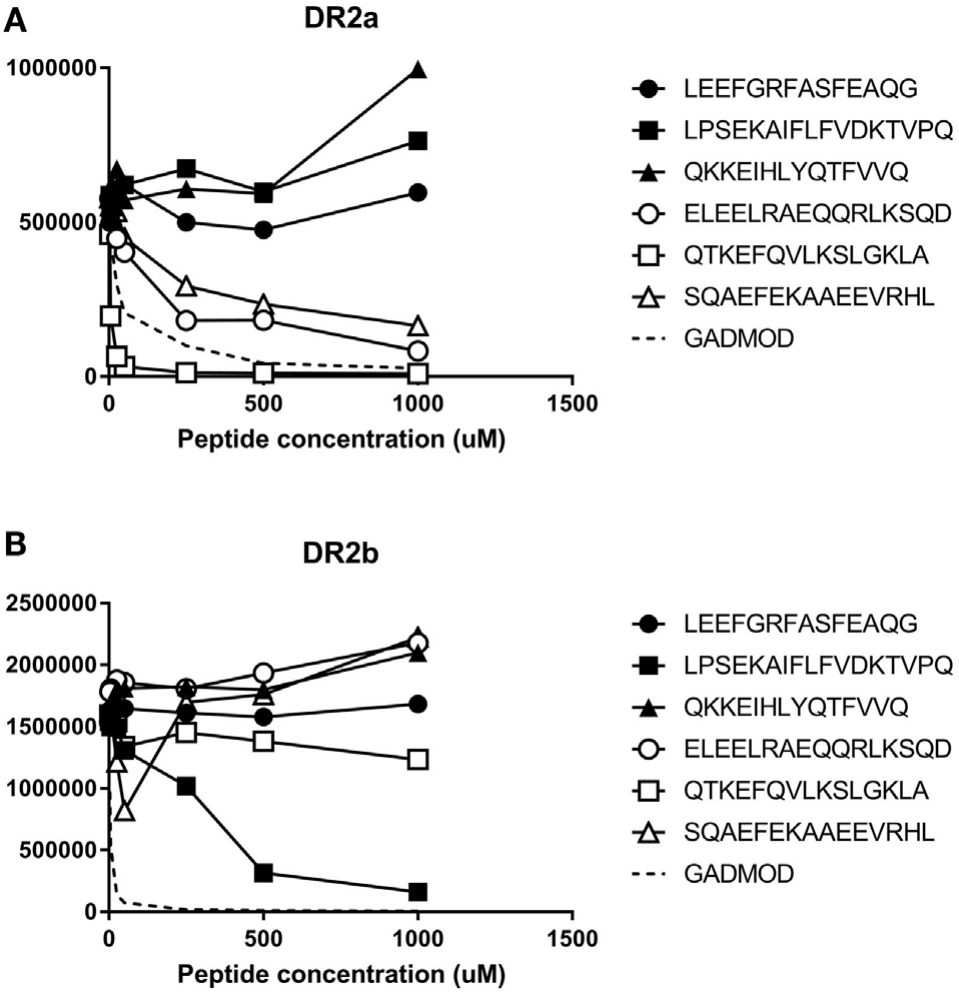
Experimental binding affinity of peptides sequenced from MGAR and **(A)** Binding to DR2a or **(B)** Binding to DR2b. Increased concentration of the peptides were incubated in wells coated with recombinant human leukocyte antigen (HLA)-DR2a or HLA-DR2b in competition with 1 µM biotinylated modified GAD555-567 peptide (NLIRVVSSNRAAT, modified from NFFRMVISNPAAT to bind to DRB1*15:01 and DRB5*01:01 in a single unambiguous register) to generate binding curves. After washing, residual biotinylated reference peptide was labeled using europium-conjugated streptavidin and quantified. The *Y*-axis depicts raw europium counts, which is proportional to the amount of residual biotinylated peptide that remains bound to HLA class II. White-filled symbols represent peptides eluted from DR2a. Black-filled symbols represent peptides eluted from DR2b.

To get an experimental measure of the binding affinities of some natural ligands of DR15, we synthesized the peptides ELEELRAEQQRLKSQDL, QTKEFQVLKSLGKLAMG, and SQAEFEKAAEEVRHL (sequenced from BLS-DR2a and the homozygous B-LCL MGAR) and the peptides LEEFGRFASFEAQG, LPSEKAIFLFVDKTVPQS, and QKKEIHLYQTFVVQ (sequenced from BLS-DR2b and MGAR). These were used in a binding assay to determine their binding affinity to DR2a and DR2b. The three peptides sequenced from DR2a showed a high affinity for DR2a ranging from 0.08 to 5.8 µM, while they failed to bind to DR2b (Figure 4A). The peptide LPSEKAIFLFVDKTVPQS, identified from DR2b, bound to DR2b with an IC50 of 14.5 µM and non-detectable affinity for DR2a. Surprisingly, the other two BLS-DR2b-sequenced peptides failed to bind to both allotypes (Figure 4B). The failure of these two peptides (with the theoretical binding cores, FGRFASFEA and IHLYQTFVV) to bind to DR2b was unexpected since they matched the DR2b motif. This was especially evident for the latter sequence, as the peptide with a high affinity harbored a theoretical core (IFLFVDKTV) with very similar main anchor residues (P1: Ile vs. Ile; P4: Tyr vs. Phe; P6: Thr vs. Asp; and P9: Val vs. Val).

### Modeling of MHC–Peptide Complexes

Computational models of the complexes of the putative cores obtained from the NetMHCIIpan 3.1 Server and some additional cores of these peptides with both DR2a and DR2b were made. The simulation protocol has been previously described (12, 30).

Table 2 shows the energetically most favored core for each peptide complexed with each molecule and the corresponding binding score (given as a Gibbs free energy). Energetically, all the peptides sequenced from DR2b would interact better with DR2b than with DR2a. The models of the peptides sequenced from DR2a indicated that four of six peptides theoretically interacted better with DR2a than with DR2b. The other two peptides bound energetically better to DR2b than to DR2a.

**Figure 5:**
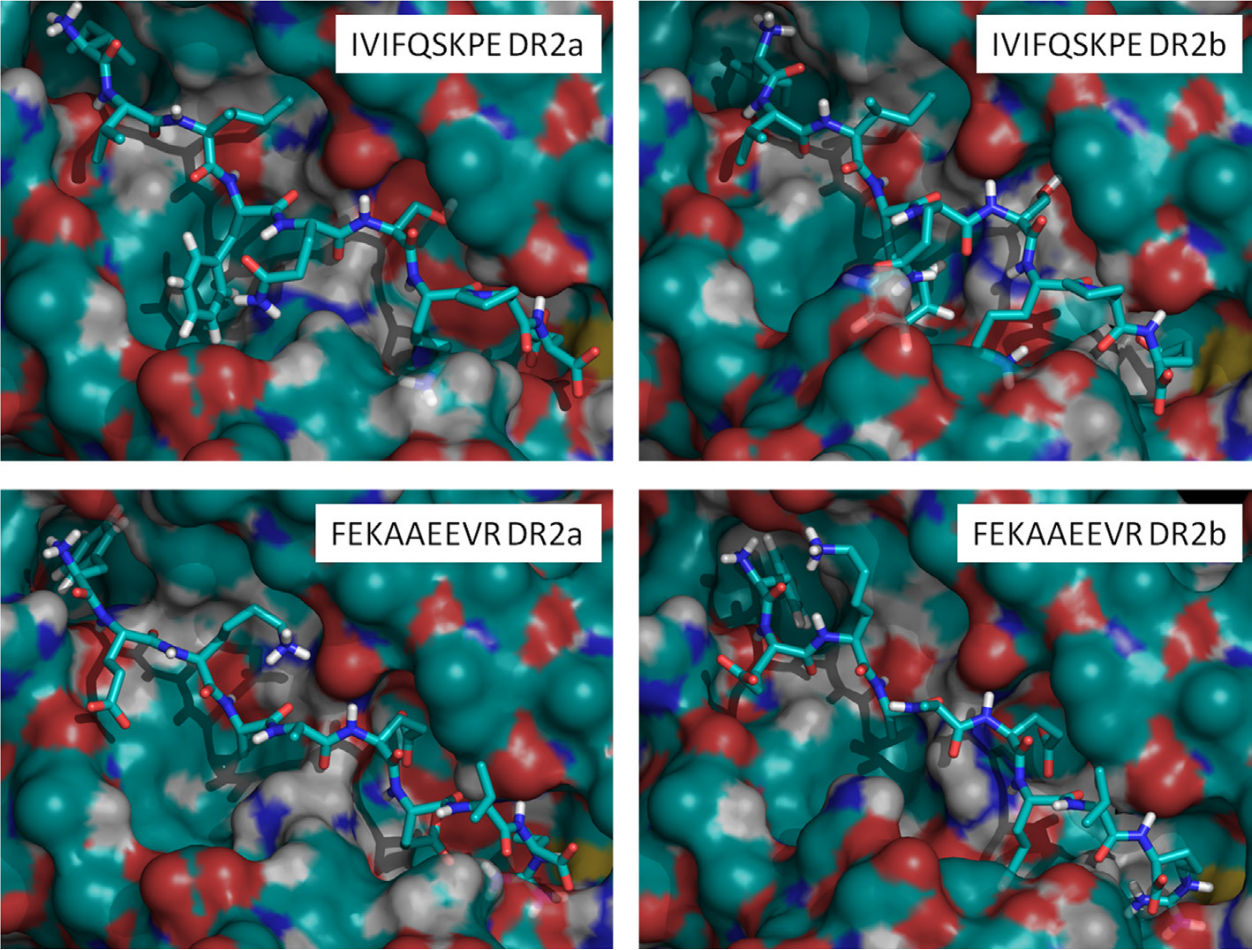
Model structures of the peptides IVIFQSKPE (eluted from DR2b) and FEKAAEEVR (eluted from DR2a) complexed with DR2a or DR2b. Computed binding scores for IVIFQSKPE are −58 kJ/mol to DR2a and −150 kJ/mol to DR2b. Computed binding scores for FEKAAEEVR are −112 kJ/mol to DR2a and −82 kJ/mol to DR2b.

Figure 5 shows examples of the cores obtained from two peptides identified in cells expressing DR2a (FEKAAEEVR) and DR2b (IVIFQSKPE) complexed with both molecules. The rest of the peptides complexed with DR2a and DR2b are shown in Figure S3 in Supplementary Material. The models shown in Figure 5 indicated that the mutation G86V reduces significantly the cavity in DR2b, sterically decreasing the affinity for the large aromatic residues. In P4, the mutation R71A increases significantly the size of the pocket in DR2b, favoring the binding of large aromatic residues. Finally, in P9 the mutations D30Y, D37S (and to a lesser extent D11P) reduce greatly the affinity of the pocket for positively charged residues in DR2b, while at the same time the mutation Q9W reduces also the size of the cavity, thus explaining the preference of this pocket in DR2b for smaller apolar residues. The peptide IVIFQSKPE showed a higher affinity for DR2b (score of −150 kJ/mol) than for DR2a (−58 kJ/mol) (Figure 5). This is consistent, according to the previous analysis, with the positioning of residues Ile in P1 (more common in DR2b), Phe in P4 (good affinity for DR2b, too bulky for DR2a) and Glu in P9 (wrong charge for DR2a, less bad for DR2b). On the other side, the peptide FEKAAEEVR shows a higher affinity for DR2a (−112 kJ/mol) than for DR2b (−82 kJ/mol). This is also consistent with the positioning of residues Phe in P1, Ala in P4, and Arg in P9, all of which with higher affinity for DR2a.

## Discussion

The HLA is polygenic, containing several different HLA genes, which encode classical HLA molecules, which are highly polymorphic and show codominant expression. These features are relevant to the function of the proteins they encode, that is, the presentation on the cell surface of peptides derived from aberrant or non-self proteins to specific T lymphocytes. In the case of HLA-II heterodimers, both chains conform the peptide-binding site. Thus, the combination of two polymorphic α chains with two polymorphic β chains, as occurs in HLA-DP and -DQ proteins, increases the theoretical capacity of peptide presentation. The fact that HLA-DRα is not polymorphic greatly reduces the diversity of the displayed peptide repertoire. However, in some haplotypes, this is compensated with the expression of two different HLA-DRB loci. The selection of these complex haplotypes may indicate that the presentation of different peptides derived from pathogens to CD4^+^ T lymphocytes constitutes a selective advantage. In addition, the presence of two different functional genes could be the result of a gene duplication that is not a disadvantage (but not necessarily an advantage) and, thus, has not been removed from the genome.

Human leukocyte antigen-DR15 is one of the haplotypes which harbors two functional loci, *DRB1* and *DRB5* typically encoding the DRB1*15:01 and DRB5*01:01 allotypes. HLA-DR15 is associated with MS, and several immunogenic peptides presented by these molecules have been reported. The anchor motifs of these molecules were described in 1994 considering the sequences of a few peptides bound to DR2a and DR2b and some binding assays (16, 17, 33). We have refined these motifs in the present work based on the peptides identified from the BLS cell line stably transfected with the corresponding genes. The resulting binding motifs are overall similar, although some clear differences emerged from our analysis. For instance, for DR2a, Ala appeared as the main anchor motif in the P4 core position, a feature that was not observed in previous reports (16, 17). A second major difference involved DR2b. In the formerly reported anchor motif, the main anchor positions were P1, P4, and P7. In our data, by contrast, these were P1, P4, P6, and P9. The P1 and P4 anchor positions were very similar to those described previously. In P6, which was not previously described as an anchor position, polar residues, principally Asn and Ser, were favored with our data. In P9, Val was the most preferred residue, but Ala and Leu were other favored residues. We did not find a clear preference for some residues in P7, which was defined as an anchor position. The peptide parental protein described here are more diverse than those described previously and locating in the endocytic compartment, what strongly suggests that most of the peptides are *bona fide* HLA-DR ligands. Since these are the canonical anchor residues in most HLA-DR molecules and we used a larger set of peptides to fine-map the DR2b binding motif, we reckon that the here reported motif is more accurate. Several crystal structures demonstrated that these are, in fact, the residues that interact with the groove both in DR2b (34, 35) and DR2a (36, 37). Moreover, the residues preferred at P6 were polar or small as described previously for most HLA-DR molecules (38).

Interestingly, P8 showed an increased frequency of positively charged residues (Lys in DR2a and Lys, His and Arg in DR2b). In this regard, we made a similar observation in DRB1*01:01, DRB1*04:01, and DRB1*10:01 (12). On the whole, our data reflect that both DR2a and DR2b favored the binding of peptides with basic residues at P8 and suggest that the preference of positively charged residues in this position may be an extended preference for many HLA-DR allotypes.

A relevant aspect that we also evaluated in this study is the promiscuity of peptide binding in HLA-II molecules. It is well-known that some peptides can bind to many HLA-DR molecules. In the case of HLA-DR15, the anchor motifs of DR2a and DR2b are different, both allotypes can accommodate similar or identical residues at major anchor positions. Thus, although with different prevalence, both molecules can accept aliphatic or aromatic residues in P1 and P4, polar residues in P6 and aliphatic and basic residues in P9. Nevertheless, our data show that, although some particular peptides can be presented by both molecules, the global degree of overlap is low. These data agree with our previous finding that, the overlap between the peptide repertoires associated with DRB1*01:01, DRB1*04:01, DRB1*10:01, and DR15 is low, even between the most similar DR1 and DR10 repertoires (12). Notably, in the here-reported datasets, the peptide overlap is even lower.

Among the peptides identified in this study, only 13 peptides were common to DR2a and DR2b. Six of them derived from the invariant chain, mirroring the high abundance of Ii-derived ligands in this cell line, a phenomenon particularly evident in DR2a. The seven remaining common peptides derived from five different proteins, two of them located in the plasma membrane, two in the nucleus, and one in the cytosol. The three peptides derived from nuclear or cytosolic proteins presented a low theoretical affinity calculated either with NetMHCIIpan 3.1 (Tables S3 and S4 in Supplementary Material) or with the matrix generated in this work (Tables S5 and S6 in Supplementary Material). Thus, we cannot discard that some of the sequenced peptides are contaminants. Nevertheless, this will not be the case for most of them as: (1) the anchor motif that we observe is similar to that described previously and (2) the subcellular locations of their parental proteins is the expected for the identified ligands. However, considering that background contaminants are more likely to be detected in both cell lines as they are allele independent, the actual overlap could be even lower than reported here.

The low degree of overlap suggests that, at least in the case of DR15, the existence of two DR alleles can increase the diversity of the peptide repertoire presented to T lymphocytes. Moreover, since some peptides can be presented in different registers, as demonstrated previously for the peptide spanning residues 86–105 of the myelin basic protein (37). In this case, the anchor residues were Phe1, Ile4, Thr6, and Thr9 for DR2a and Val1, Phe4, Asn6, and Thr for DR2b, which are favored or well-accepted residues in the binding motifs described in this report. The presentation of the same peptide in different registers allows to increase the number of CD4^+^ T cells recognizing a particular antigenic peptide as different clones would recognize the different registers of each peptides.

The modeled structures of the peptide ligands complexed with DR2a and DR2b can be rationalized in light of the anchor motifs obtained in this work. Thus, the binding motifs for both alleles show that selection is primarily done at the level of P1, P4, and P9. P1 binds preferentially large aromatic residues in DR2a and smaller aliphatic residues (preferentially Ile) in DR2b. P4 shows the opposite trend, with small aliphatic residues in DR2a and aromatic residues in DR2b. Finally, P9 shows a preference for basic residues in DR2a and aliphatic residues in DR2b. These preferences can be broadly explained by the sequence differences between DR2a and DR2b. Thus, the dimorphism G86V explains the preference of each allotype in P1. As seen in Figure 3, P4 is a more restrictive position in DR2b than in DR2a, what can be structurally explained by the polymorphism R71A (Figure 5). P9 is more restrictive in DR2a than in DR2b, having preference for basic residues, what is explained by the presence of Asp30, Asp37, and Asp11. Although the conclusions of *in silico* models are speculative, there are data enough in the literature that experimentally confirm our main conclusions. Specifically, the mutant G86V is a natural polymorphism present in some HLA-DR alleles. Thus, many of the most studied alleles contain Gly86, while DRB1*03:01 and DRB1*04:04 contain Val86. We analyzed the peptidomes bound to some HLA-DR molecules containing Gly86, as DRB1*01:01 (12, 24, 39) and all of them bind preferentially peptides with aromatic residues in P1. HLA-DRB1*04:01 present Gly86 while HLA-DRB1*04:02 and HLA-DRB1*04:04 contain Val86. The binding motifs of these allotypes have been described using transfectants where the DRB4 gene was not expressed (40). DRB1*04:01 also binds principally Phe and Tyr, while the two molecules with Val86 prefer smaller aliphatic residues. Thus, these data confirm our pMHC computer-based models. Regarding the P4 anchor position, there are other polymorphisms in the residues affecting the corresponding pocket and there are few common DRB genes with Ala71. Nevertheless, some studied HLA-DR molecules contain Arg71, as DRB1*04:04, DRB1*08:01, DRB1*10:01, and all of them bind preferentially aliphatic residues in P4, in concordance with the residues found for DRB5*01:01 (which also contains Arg71) and in contrast with the clear preference for aromatic residues obtained for DRB1*15:01 (which contains Ala71). Finally, regarding the DRB residues proposed to affect the interaction with the P9 anchor residue, there are not common alleles carrying Asp in these positions. However, the conclusion that the preference for basic residues in P9 is due to the introduction of three negative charges (P11D, Y30D, and S37D) in DR2a is very likely, as indicated by the models.

In this work, we have used an LC–MS/MS-based peptidomic approach to refine the binding model of two HLA-DR allotypes associated with MS. To our knowledge, this is the first time that the peptide repertoires of two HLA-DR molecules belonging to the same haplotype are directly compared. In this context, our data indicate that the overlap in their bound peptidomes is very low. In addition, our data suggest that, quantitatively both molecules contribute to a similar extent to the configuration of the peptide pool displayed by HLA-DR15 on the cell surface. Finally, although the peptides sequenced here do not have special relevance to MS, as they do not come from known autoantigens, we propose that the analysis of peptide ligands eluted from HLA-DR molecules shows to be complementary to bioinformatics tools and can contribute to improve the prediction of new T cell epitopes relevant for autoimmune diseases.

## Author Contributions

ES contributed with drafting the work and the acquisition, analysis, and interpretation of the data. MM contributed with the acquisition of mass spectrometry data. XD contributed with the computer models. DA-L and EJ contributed with the acquisition and interpretation of binding assays. IA contributed with drafting the work, interpretation of the data, revising, and final approval of the version published.

## Conflict of Interest Statement

The authors declare that the research was conducted in the absence of any commercial or financial relationships that could be construed as a potential conflict of interest.

## Supplementary Material

Figure S1 SDS-PAGE gel of immunoprecipitated HLA-DR2a and -DR2b molecules. Peptide–HLA-DR complexes were eluted in acid medium and passed through a Centricon-10 device. The retained material was loaded on a 12% SDS-PAGE gel. Arrows show α and β HLA-DR chains.

Figure S2 Total ion chromatogram and fragmentation spectra of peptides sequenced from DR2a and DR2b molecules. Pages 1, 2, and 6 show the total ion chromatograms (TICs) of a blank, DR2a and DR2b samples, respectively. Pages 3 and 7 show the extracted ion chromatograms (XICs) of signals 790.9301 (from DR2a) and 746.0306 (from DR2a), respectively. Page 4 shows the fragmentation spectrum of the peptide TPLLMQALPMGALPQ, and page 5 shows the annotation of the corresponding fragments (black fonts correspond to the theoretical masses generated during the peptide fragmentation; red fonts correspond to the masses found in the fragmentation spectrum). Page 8 shows the fragmentation spectrum of the peptide GLQADLSSFKSQELNERNEA, and page 9 shows the annotation of the corresponding fragments (black fonts correspond to the theoretical masses generated during the peptide fragmentation; red fonts correspond to the masses found in the fragmentation spectrum).

Figure S3 Model structures of DR15 ligands complexed with DR2a and DR2b. The interactions of the identified binding cores from peptides sequenced from MGAR (DR15) and from BLS-DR2a or -DR2b and DR2a and DR2b were modeled. Figures show the graphical representation of the models most favorable energetically.
