# Supplementary figures and images for: Human Leukocyte Antigen (HLA)-DRB1*15:01 and HLA-DRB5*01:01 Present Complementary Peptide Repertoires
Source: Front Immunol. 2017 Aug 21;8:984. doi: 10.3389/fimmu.2017.00984 (PMC5566978; doi:10.3389/fimmu.2017.00984)

## GEL SDS-PAGE 12%

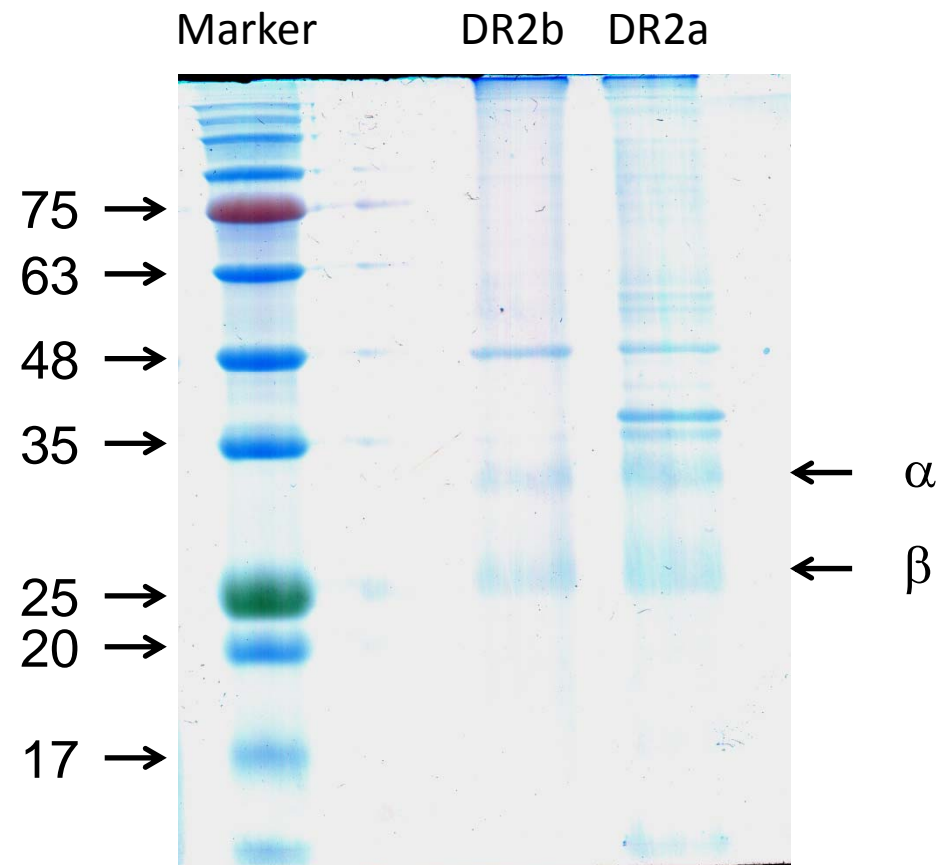

Supplement: Figure S1 — SDS-PAGE gel of immunoprecipitated HLA-DR2a and -DR2b molecules. Peptide–HLA-DR complexes were eluted in acid medium and passed through a Centricon-10 device. The retained material was loaded on a 12% SDS-PAGE gel. Arrows show α and β HLA-DR chains. [file Image_1.PDF]
