# Supplementary material for: Human Leukocyte Antigen (HLA)-DRB1*15:01 and HLA-DRB5*01:01 Present Complementary Peptide Repertoires
Source: Front Immunol. 2017 Aug 21;8:984. doi: 10.3389/fimmu.2017.00984 (PMC5566978; doi:10.3389/fimmu.2017.00984)
Supplement: Figure S2 — Total ion chromatogram and fragmentation spectra of peptides sequenced from DR2a and DR2b molecules. Pages 1, 2, and 6 show the total ion chromatograms (TICs) of a blank, DR2a and DR2b samples, respectively. Pages 3 and 7 show the extracted ion chromatograms (XICs) of signals 790.9301 (from DR2a) and 746.0306 (from DR2a), respectively. Page 4 shows the fragmentation spectrum of the peptide TPLLMQALPMGALPQ, and page 5 shows the annotation of the corresponding fragments (black fonts correspond to the theoretical masses generated during the peptide fragmentation; red fonts correspond to the masses found in the fragmentation spectrum). Page 8 shows the fragmentation spectrum of the peptide GLQADLSSFKSQELNERNEA, and page 9 shows the annotation of the corresponding fragments (black fonts correspond to the theoretical masses generated during the peptide fragmentation; red fonts correspond to the masses found in the fragmentation spectrum). [file Image_2.PDF]

TIC Blank (MS only)

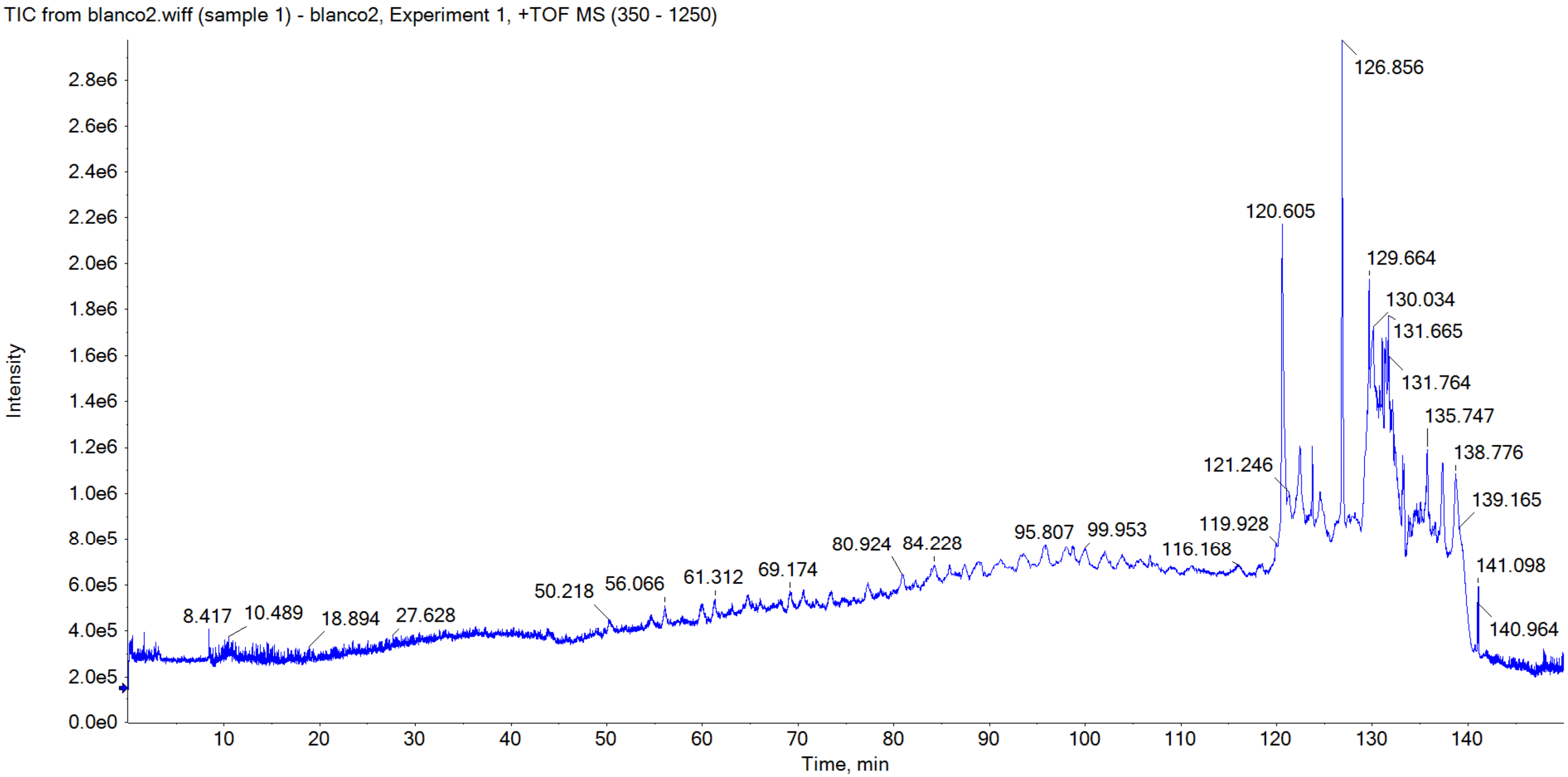

TIC DR2a (MS only)

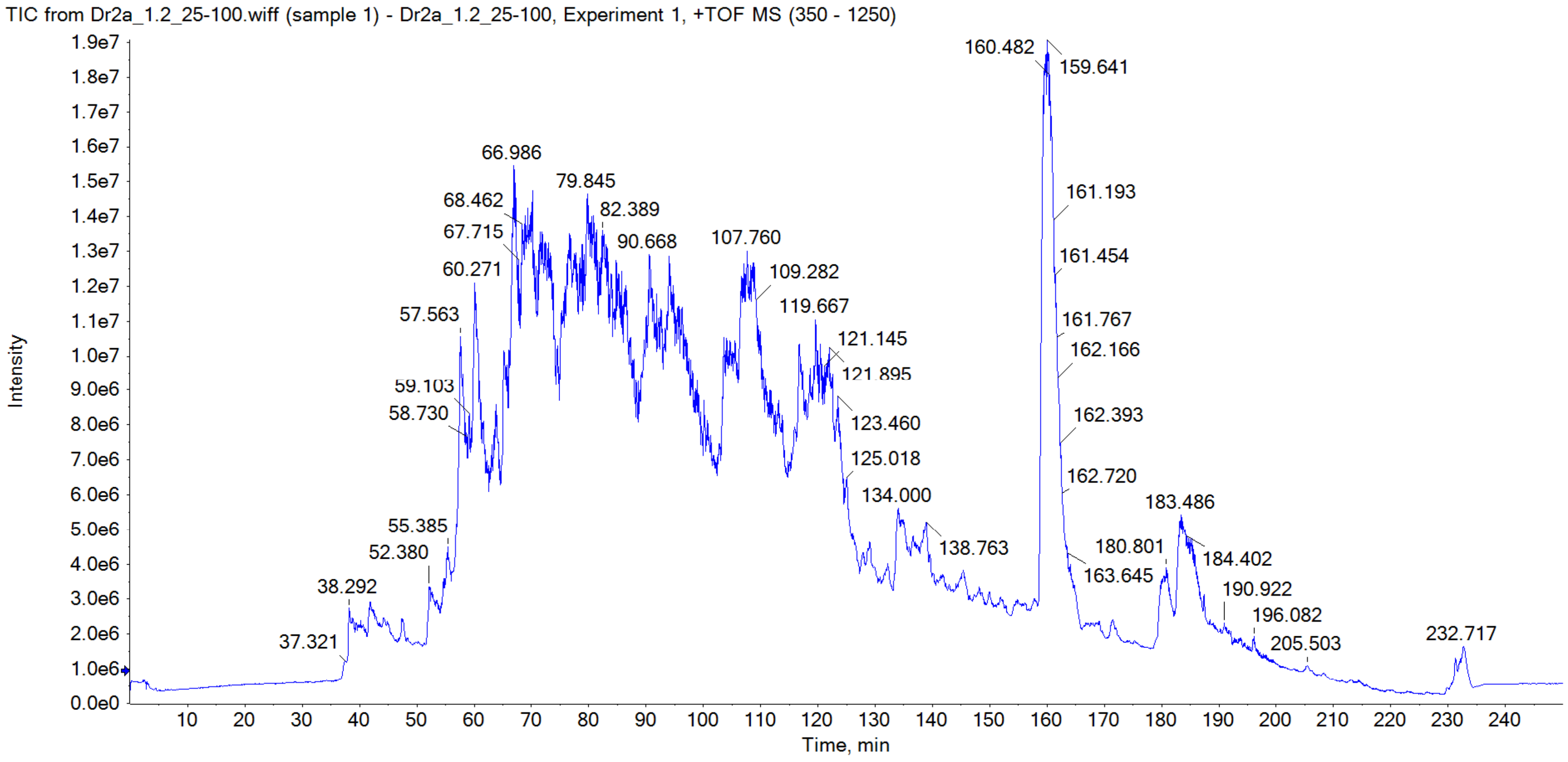

DR2a – XIC (790.9301)

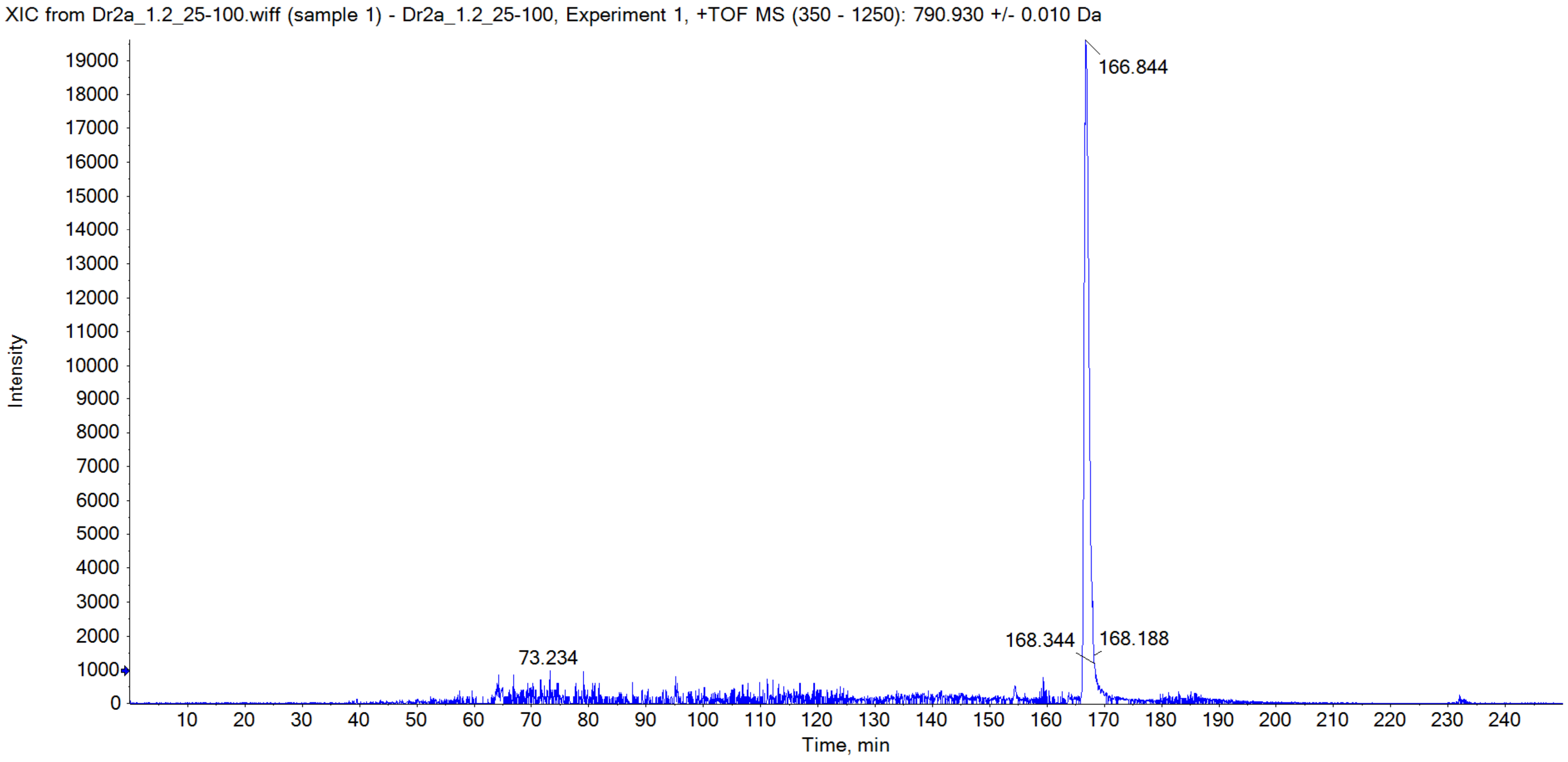

MS2 (790.9301/2+)
TPLLMQALPMGALPQ

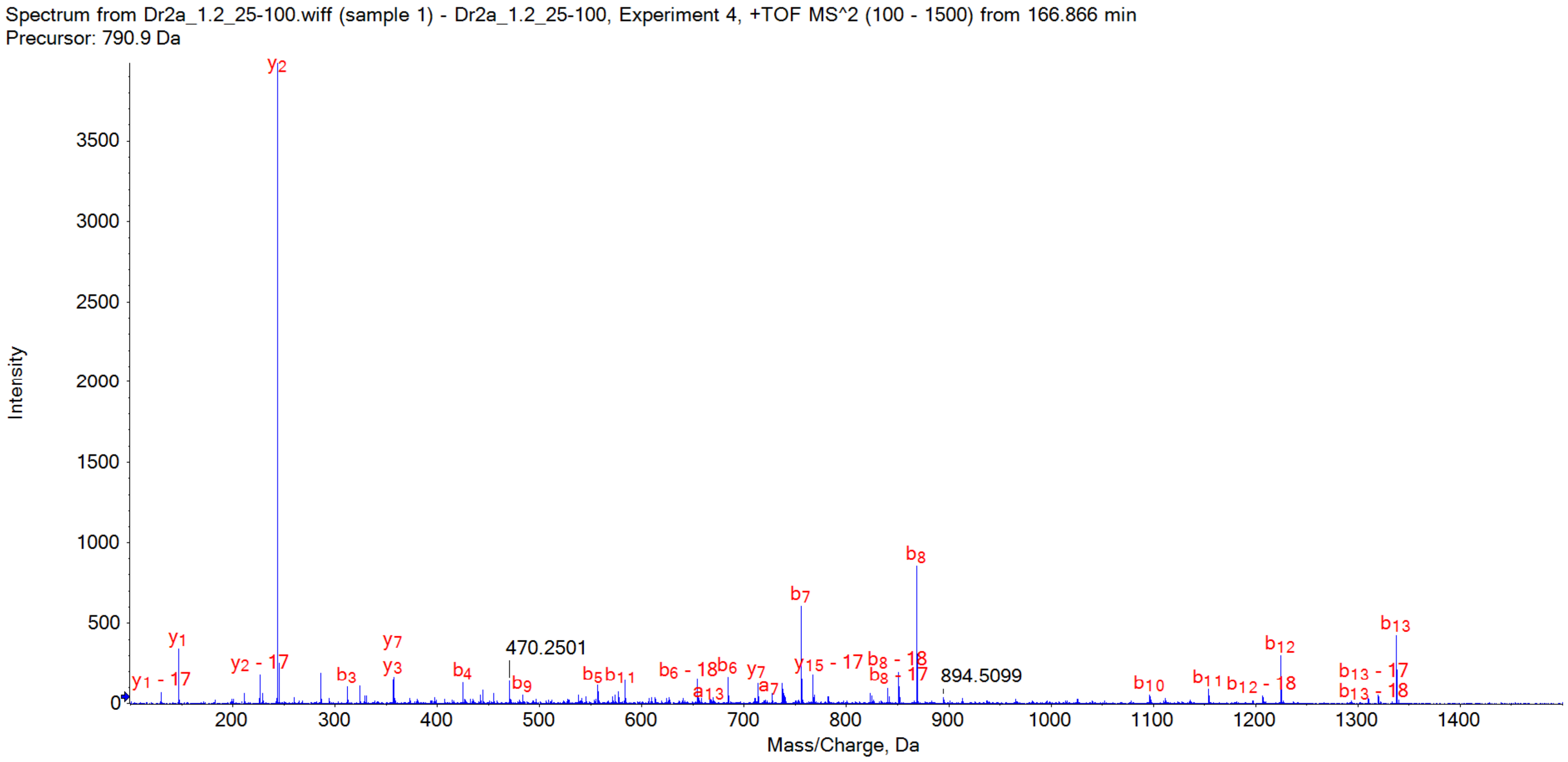

MS2 (790.9301/2+)

TPLLMQALPMGALPQ

Symbol	Res. Mass	# (N)	a	a - 17	a - 18	b	b - 17	b - 18	y	y - 17	y - 18	# (C)
T	101.04768	1	74.06004	57.03349	56.04948	102.05496	85.02841	84.04439	1580.85389	*1563.82734*	1562.84332	15
P	97.05276	2	171.11280	154.08626	153.10224	199.10772	182.08117	181.09715	1479.80621	1462.77966	1461.79564	14
L	113.08406	3	284.19687	267.17032	266.18630	**312.19178**	295.16523	294.18122	1382.75344	1365.72689	1364.74288	13
L	113.08406	4	397.28093	380.25438	379.27037	**425.27585**	408.24930	407.26528	1269.66938	1252.64283	1251.65881	12
M	131.04049	5	528.32142	511.29487	510.31085	**556.31633**	539.28978	**538.30577**	1156.58532	1139.55877	1138.57475	11
Q	128.05858	6	656.38000	639.35345	638.36943	**684.37491**	667.34836	**666.36435**	1025.54483	1008.51828	1007.53426	10
A	71.03711	7	**727.41711**	710.39056	709.40655	**755.41202**	**738.38548**	**737.40146**	897.48625	880.45970	879.47569	9
L	113.08406	8	**840.50117**	**823.47462**	822.49061	**868.49609**	**851.46954**	**850.48552**	826.44914	809.42259	808.43857	8
P	97.05276	9	937.55394	920.52739	919.54337	*965.54885*	948.52230	947.53829	***713.36507***	696.33853	695.35451	7
M	131.04049	10	1068.59442	1051.56787	1050.58386	**1096.58934**	1079.56279	1078.57877	616.31231	599.28576	598.30175	6
G	57.02146	11	1125.61589	1108.58934	1107.60532	***1153.61080***	1136.58425	1135.60024	485.27182	468.24528	467.26126	5
A	71.03711	12	1196.65300	1179.62645	1178.64244	**1224.64792**	1207.62137	**1206.63735**	428.25036	411.22381	410.23980	4
L	113.08406	13	*1309.73707*	1292.71052	1291.72650	**1337.73198**	**1320.70543**	**1319.72142**	**357.21325**	340.18670	339.20268	3
P	97.05276	14	1406.78983	1389.76328	1388.77926	1434.78474	1417.75819	1416.77418	**244.12918**	**227.10263**	226.11862	2
Q	128.05858	15	1534.84841	1517.82186	1516.83784	1562.84332	1545.81677	1544.83276	**147.07642**	**130.04987**	129.06585	1

TIC DR2b (MS only)

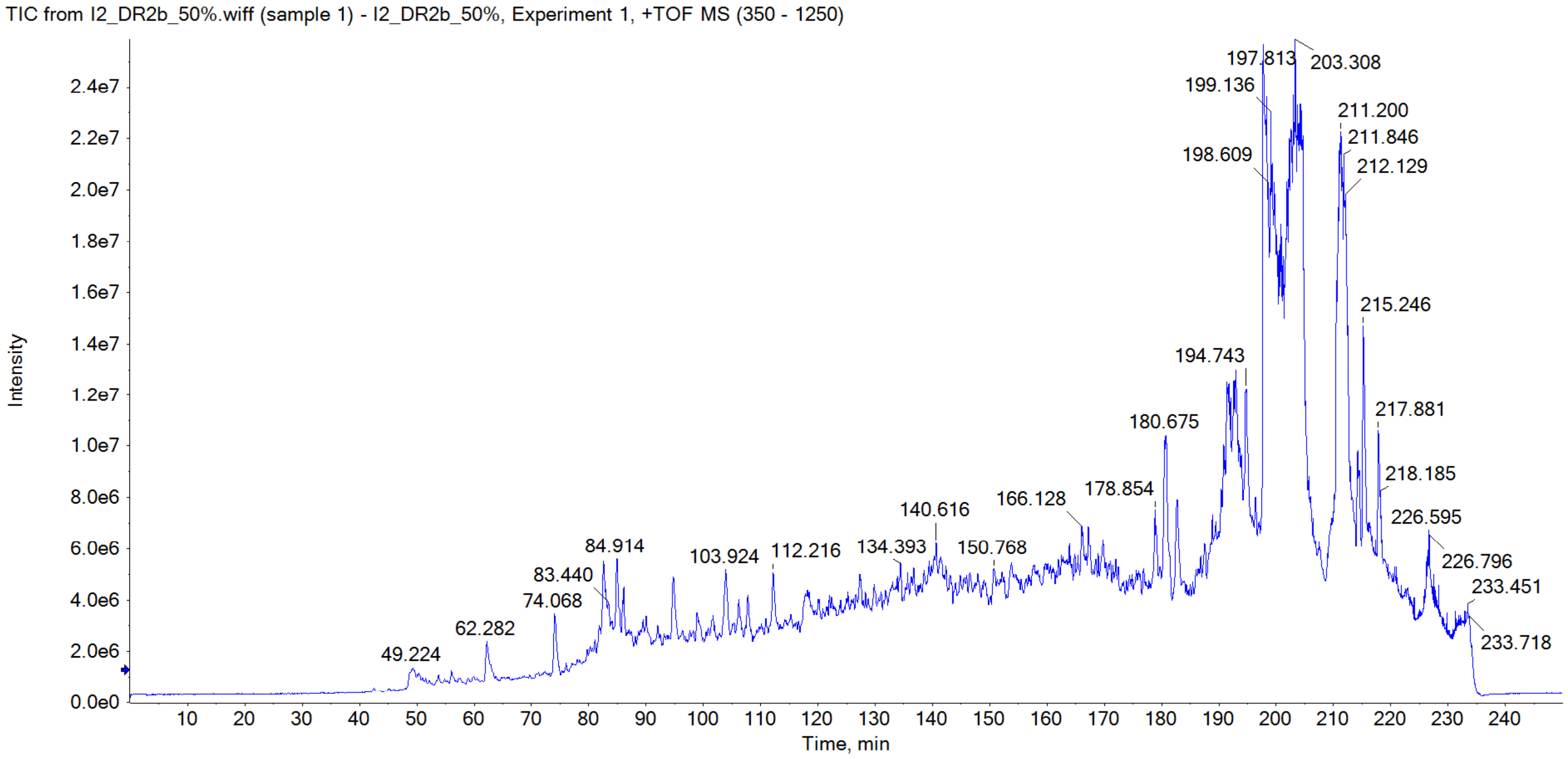

DR2b – XIC (746.0306)

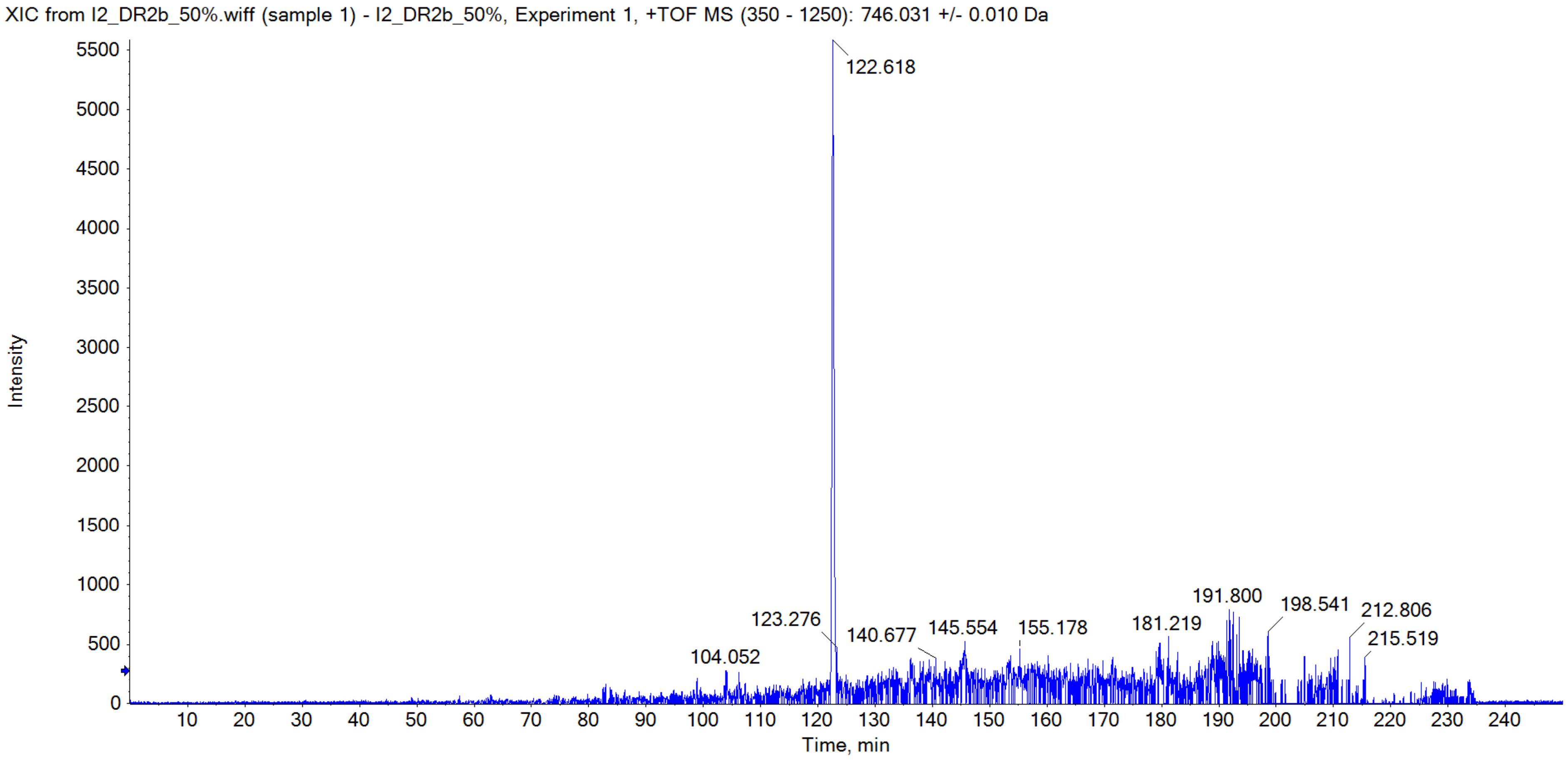

MS2 (746.0306 /3+)

GLQADLSSFKSQELNERNEA

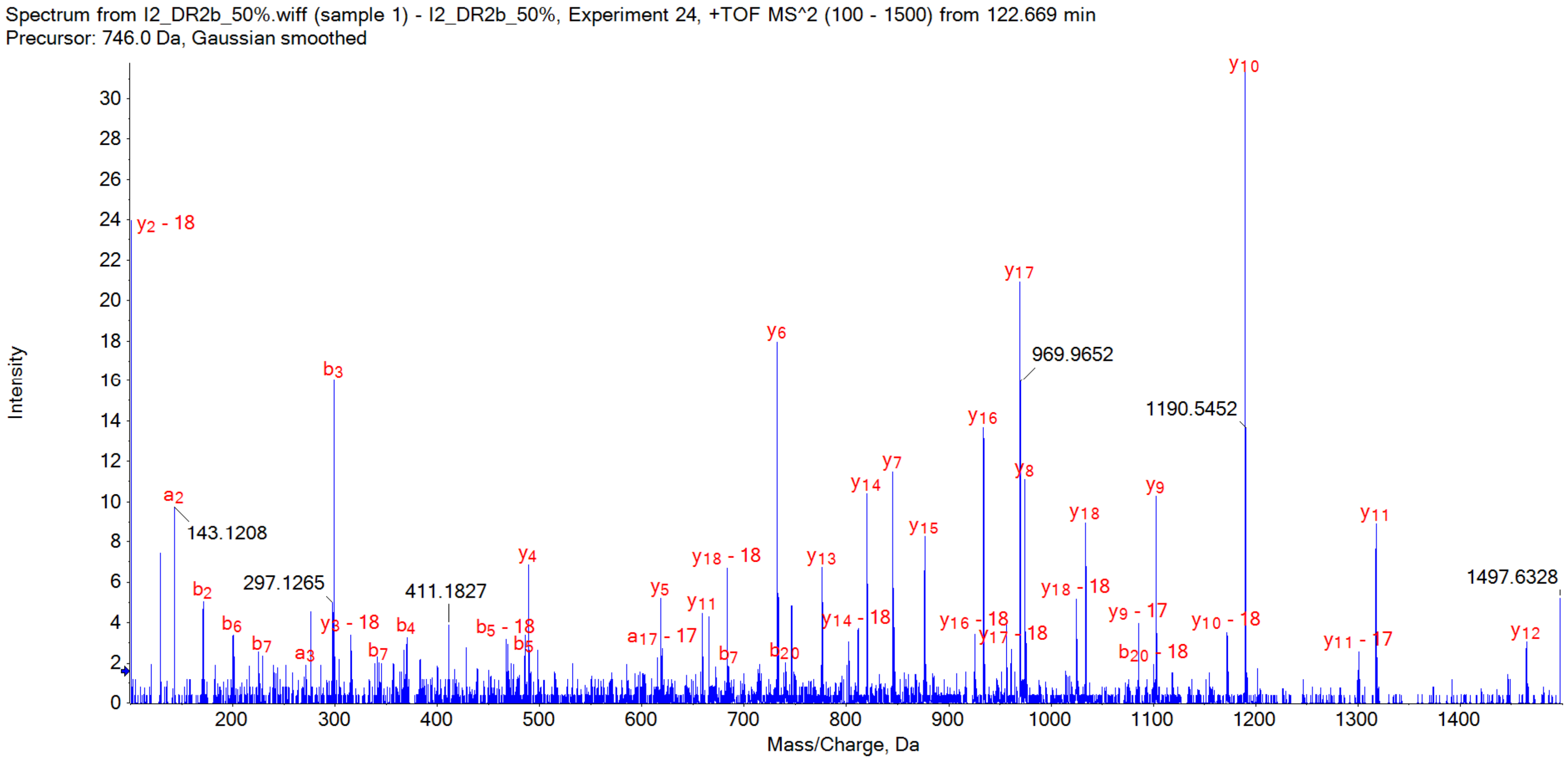

MS2 (746.0306 /3+)

GLQADLSSFKSQELNERNEA

Symbol	Res. Mass	# (N)	a	a - 17	a - 18	b	b - 17	b - 18	y	y - 17	y - 18	# (C)
G	57.02146	1	30.03383	13.00728	12.02326	58.02874	41.00219	40.01818	2236.08403	2219.05748	*2218.07347*	20
L	113.08406	2	**143.11789**	126.09134	125.10732	**171.11280**	154.08626	153.10224	2179.06257	2162.03602	2161.05200	19
Q	128.05858	3	**271.17647**	254.14992	253.16590	**299.17138**	282.14483	281.16082	*2065.97850*	*2048.95195*	*2047.96794*	18
A	71.03711	4	342.21358	325.18703	324.20302	**370.20850**	353.18195	352.19793	*1937.91993*	*1920.89338*	*1919.90936*	17
D	115.02694	5	*457.24052*	440.21398	**439.22996**	**485.23544**	468.20889	**467.22487**	*1866.88281*	*1849.85626*	*1848.87225*	16
L	113.08406	6	570.32459	553.29804	552.31402	*598.31950*	581.29295	580.30894	*1751.85587*	1734.82932	1733.84530	15
S	87.03203	7	657.35662	640.33007	639.34605	***685.35153***	668.32498	667.34097	*1638.77181*	*1621.74526*	*1620.76124*	14
S	87.03203	8	744.38865	727.36210	726.37808	772.38356	755.35701	*754.37300*	*1551.73978*	1534.71323	1533.72921	13
F	147.06841	9	891.45706	874.43051	873.44649	919.45197	902.42542	901.44141	***1464.70775***	1447.68120	1446.69718	12
K	128.09496	10	1019.55202	1002.52547	1001.54146	1047.54694	1030.52039	1029.53637	***1317.63933***	**1300.61279**	1299.62877	11
S	87.03203	11	1106.58405	1089.55750	1088.57349	1134.57897	1117.55242	1116.56840	**1189.54437**	**1172.51782**	**1171.53381**	10
Q	128.05858	12	1234.64263	1217.61608	1216.63206	1262.63754	1245.61099	1244.62698	**1102.51234**	**1085.48579**	1084.50178	9
E	129.04259	13	1363.68522	1346.65867	1345.67466	1391.68014	1374.65359	1373.66957	**974.45376**	**957.42722**	**956.44320**	8
L	113.08406	14	1476.76929	1459.74274	1458.75872	1504.76420	1487.73765	1486.75364	**845.41117**	828.38462	827.40061	7
N	114.04293	15	1590.81221	1573.78566	1572.80165	1618.80713	1601.78058	1600.79656	**732.32711**	**715.30056**	714.31654	6
E	129.04259	16	1719.85481	1702.82826	1701.84424	1747.84972	1730.82317	1729.83916	**618.28418**	*601.25763*	600.27362	5
R	156.10111	17	1875.95592	*1858.92937*	1857.94535	1903.95083	1886.92428	1885.94027	**489.24159**	**472.21504**	471.23102	4
N	114.04293	18	1989.99885	1972.97230	1971.98828	2017.99376	2000.96721	1999.98320	333.14048	**316.11393**	**315.12991**	3
E	129.04259	19	2119.04144	2102.01489	2101.03087	2147.03635	2130.00980	2129.02579	219.09755	202.07100	***201.08698***	2
A	71.03711	20	2190.07855	2173.05200	2172.06799	*2218.07347*	2201.04692	*2200.06290*	90.05496	73.02841	72.04439	1
