# Supplementary material for: Human Leukocyte Antigen (HLA)-DRB1*15:01 and HLA-DRB5*01:01 Present Complementary Peptide Repertoires
Source: Front Immunol. 2017 Aug 21;8:984. doi: 10.3389/fimmu.2017.00984 (PMC5566978; doi:10.3389/fimmu.2017.00984)

FGRFASFEA DR2a

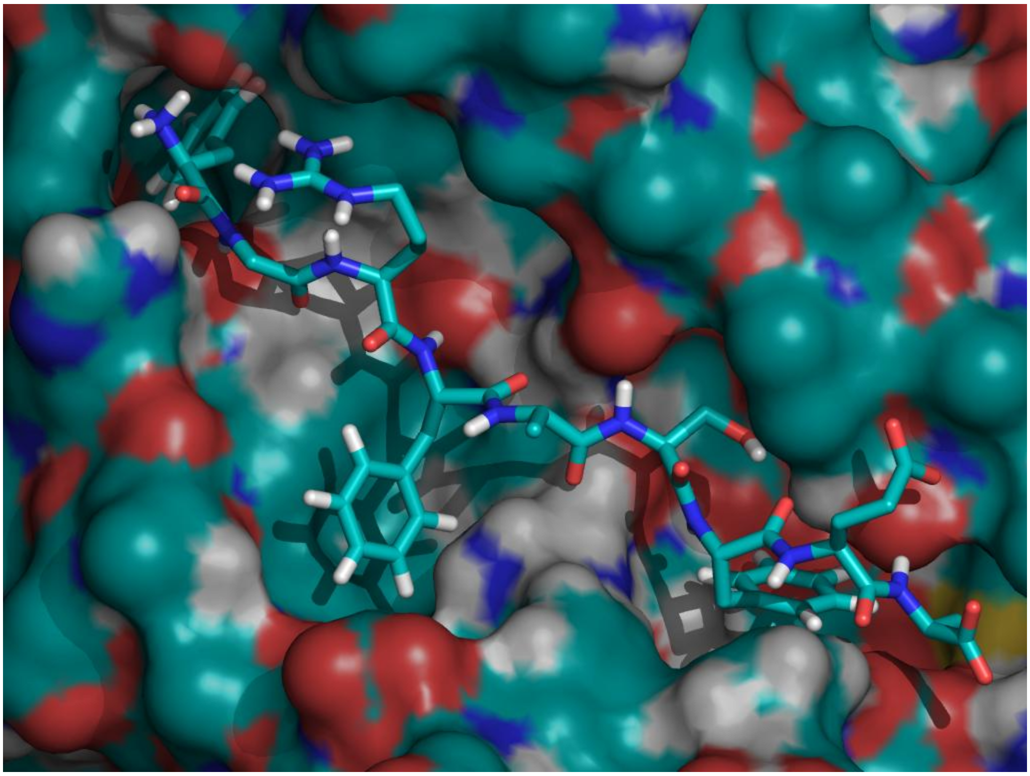

FGRFASFEA DR2b

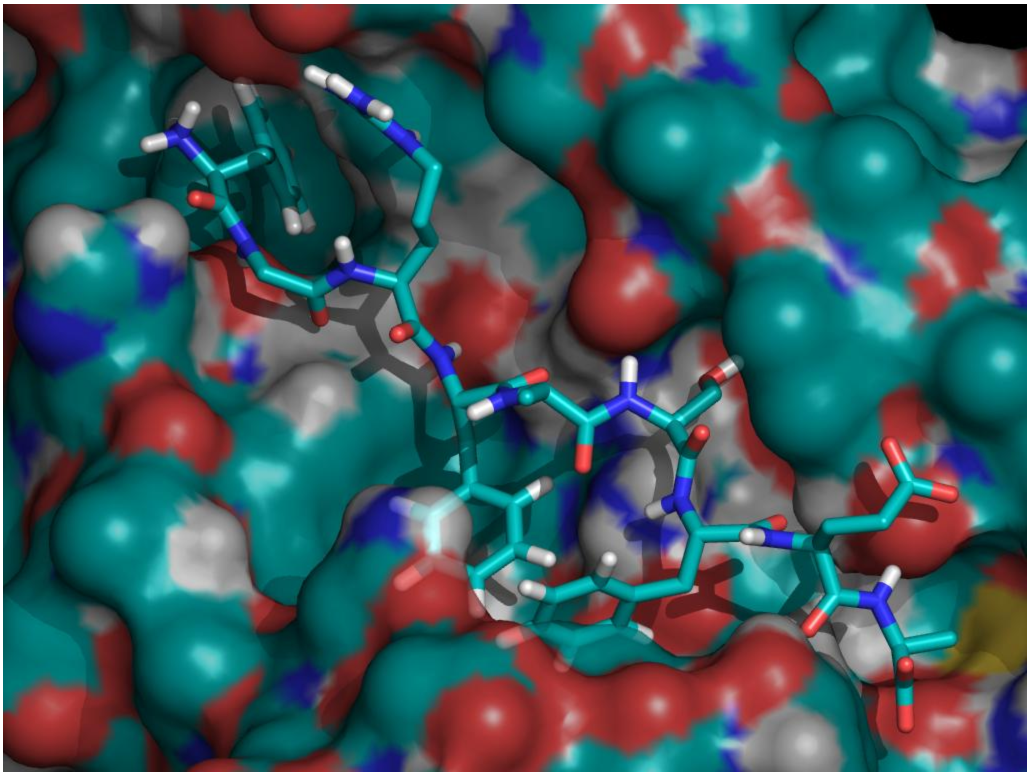

LALFPGIRL DR2a

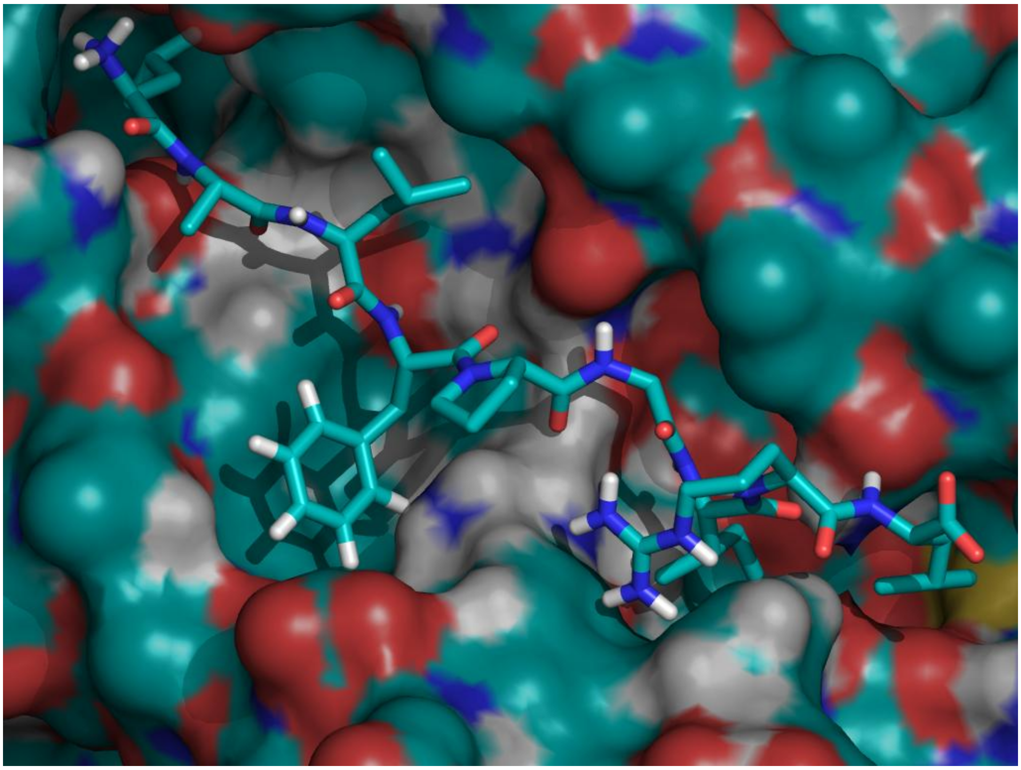

LALFPGIRL DR2b

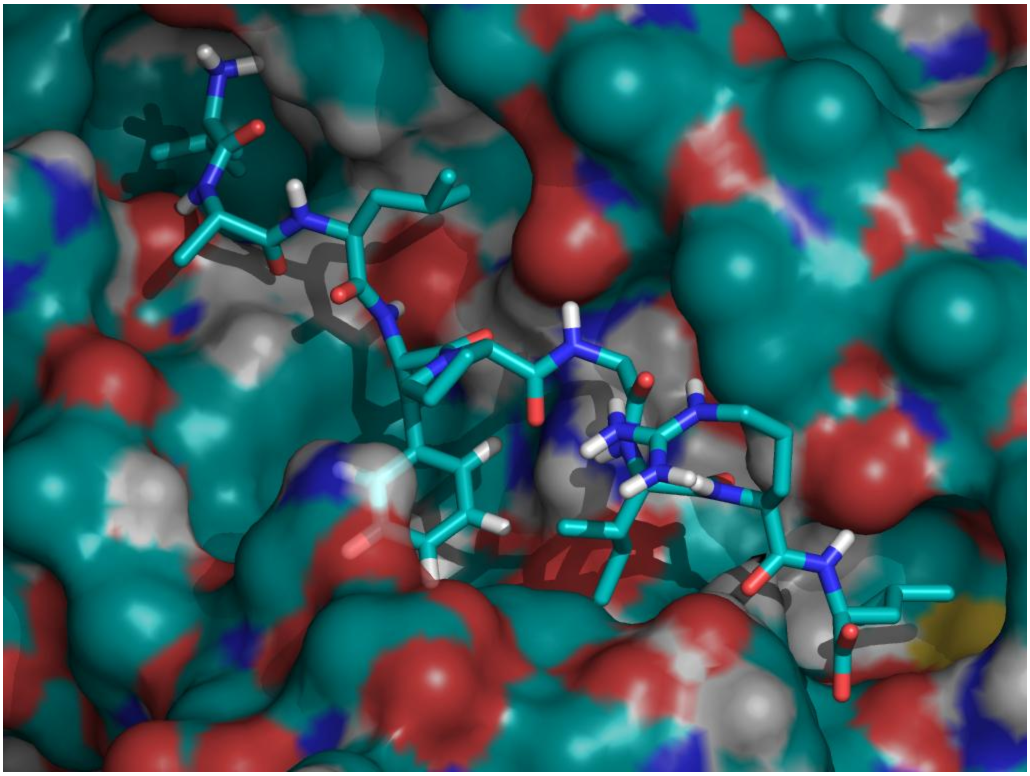

IFLFVDKTV DR2a

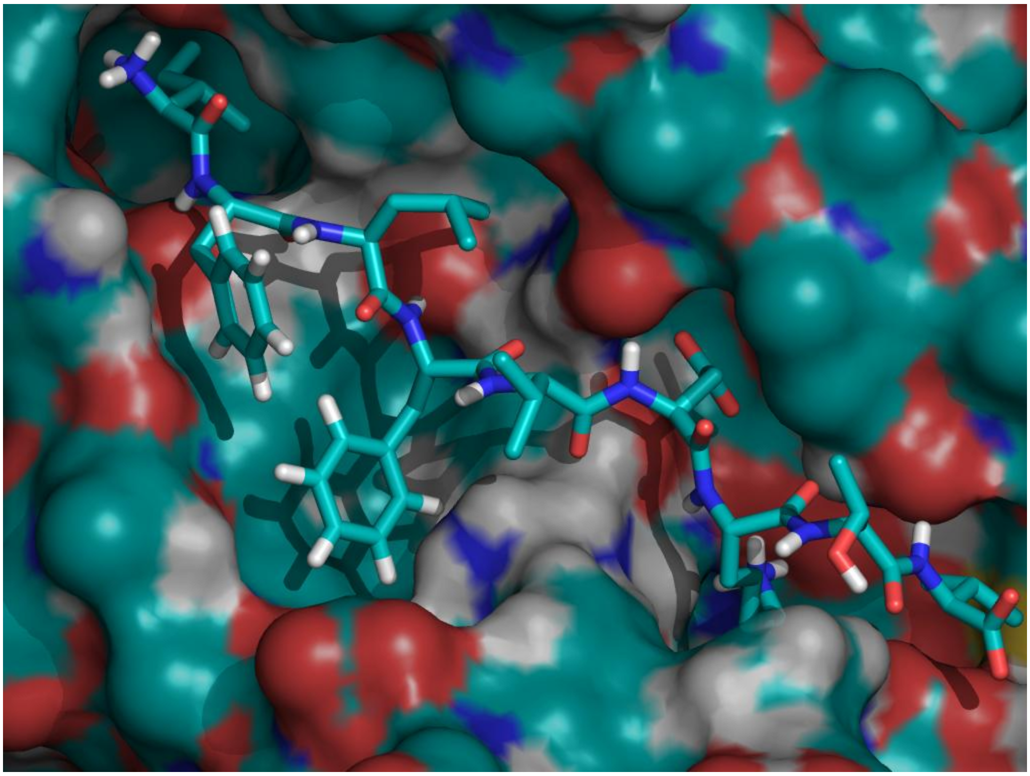

IFLFVDKTV DR2b

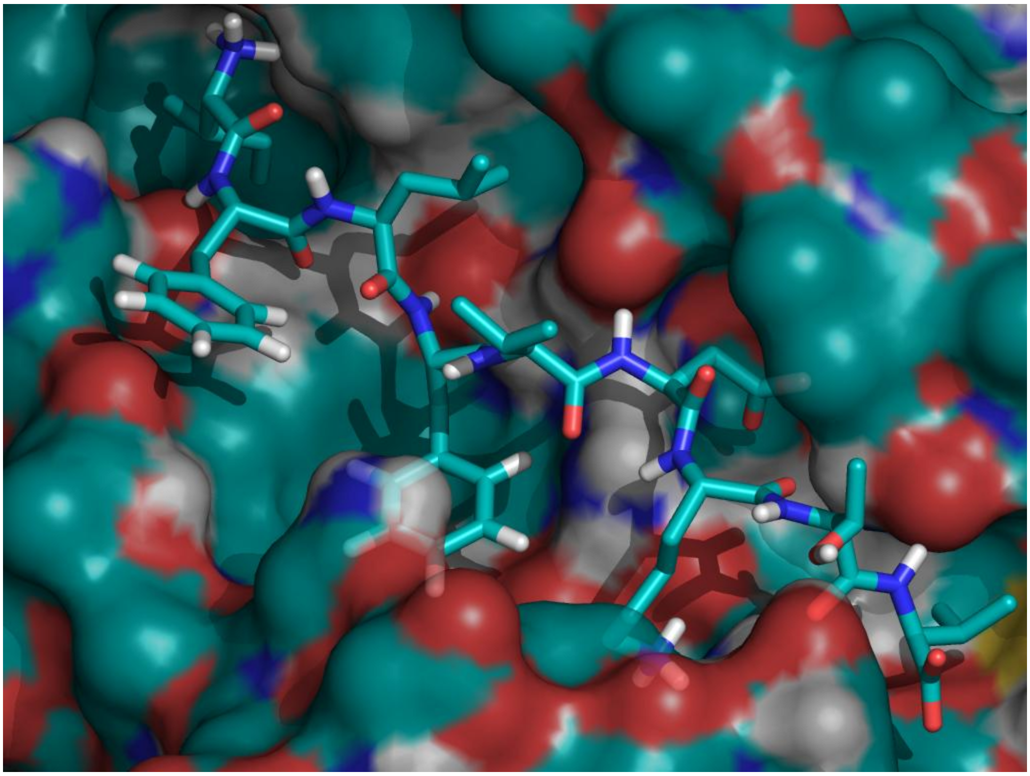

VRVVVPYQG DR2a

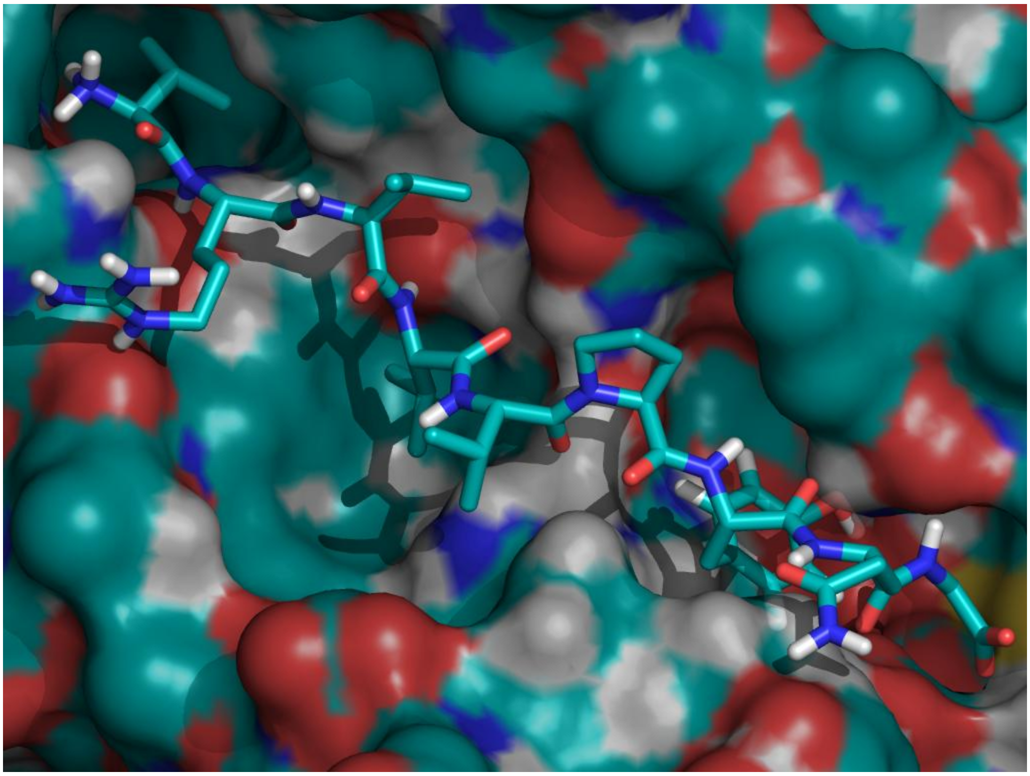

VRVVVPYQG DR2b

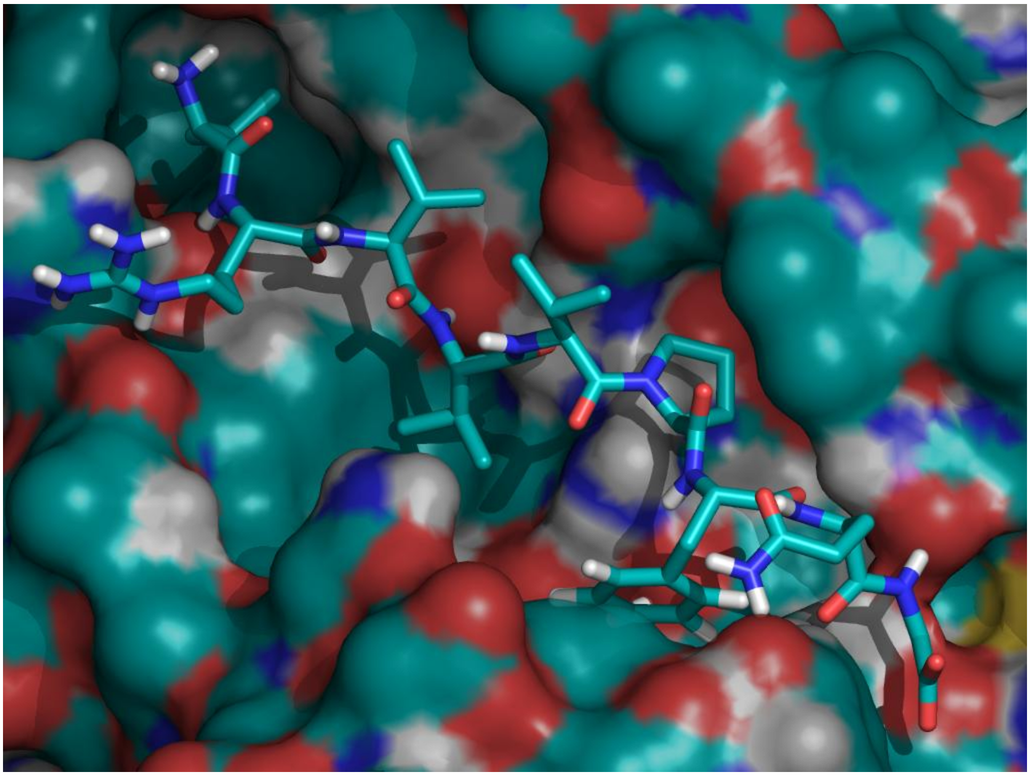

LNRYPASSL DR2a

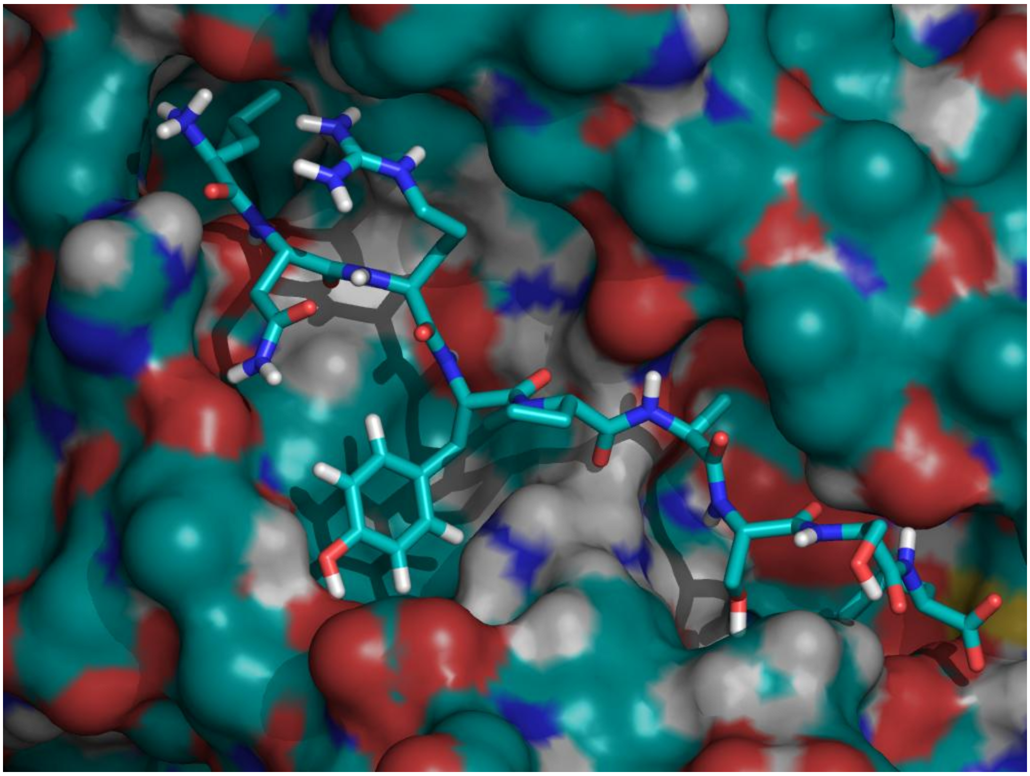

LNRYPASSL DR2b

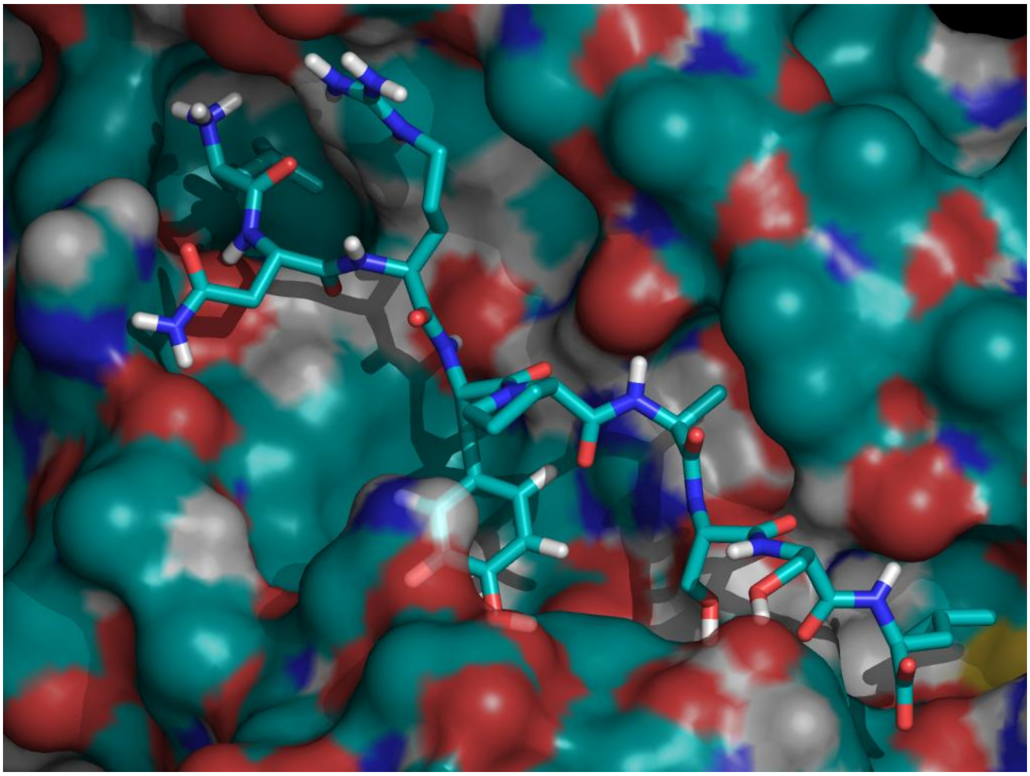

IHLYQTFVVV DR2a

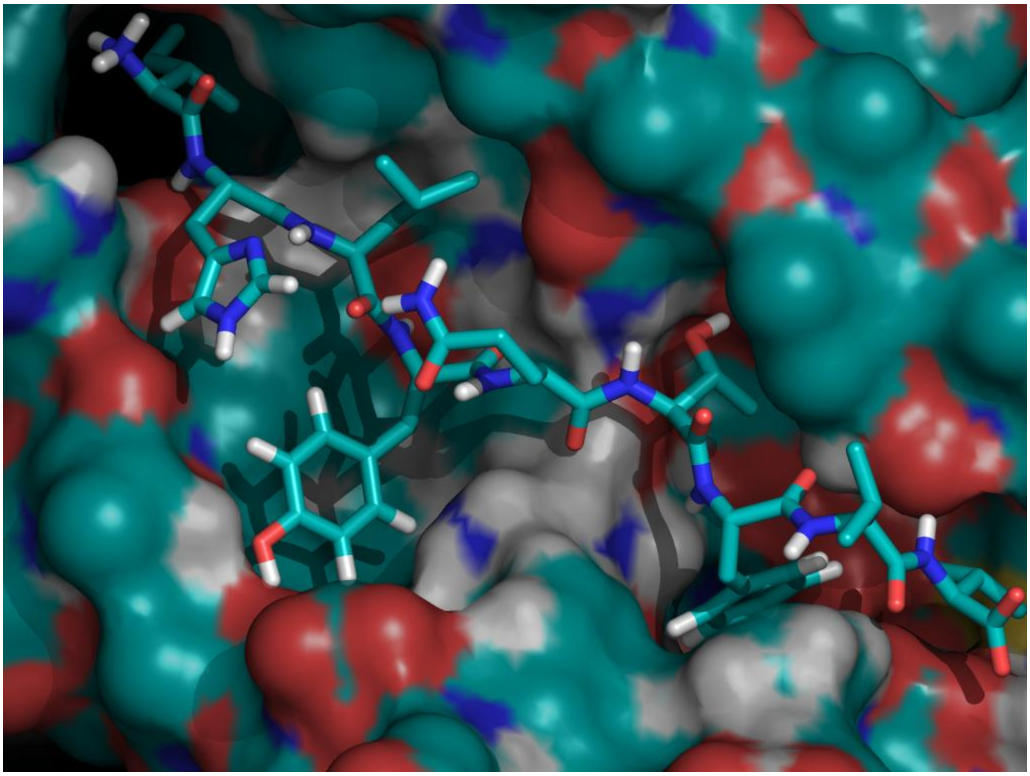

IHLYQTFVVV DR2b

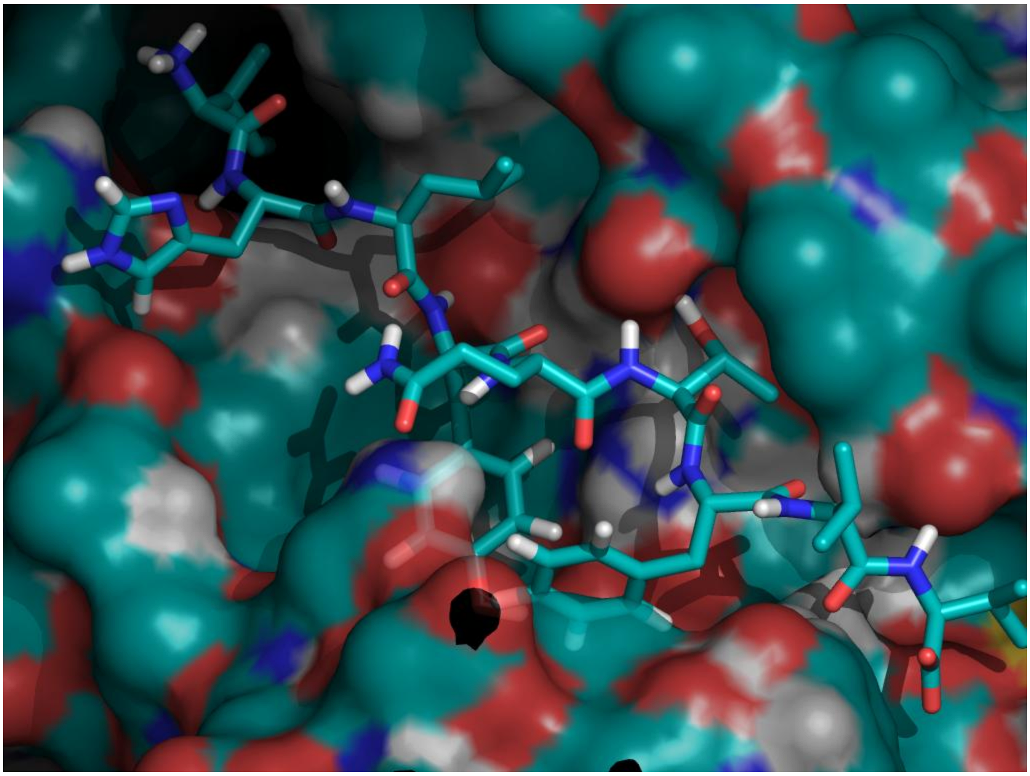

IQVYEGERA DR2a

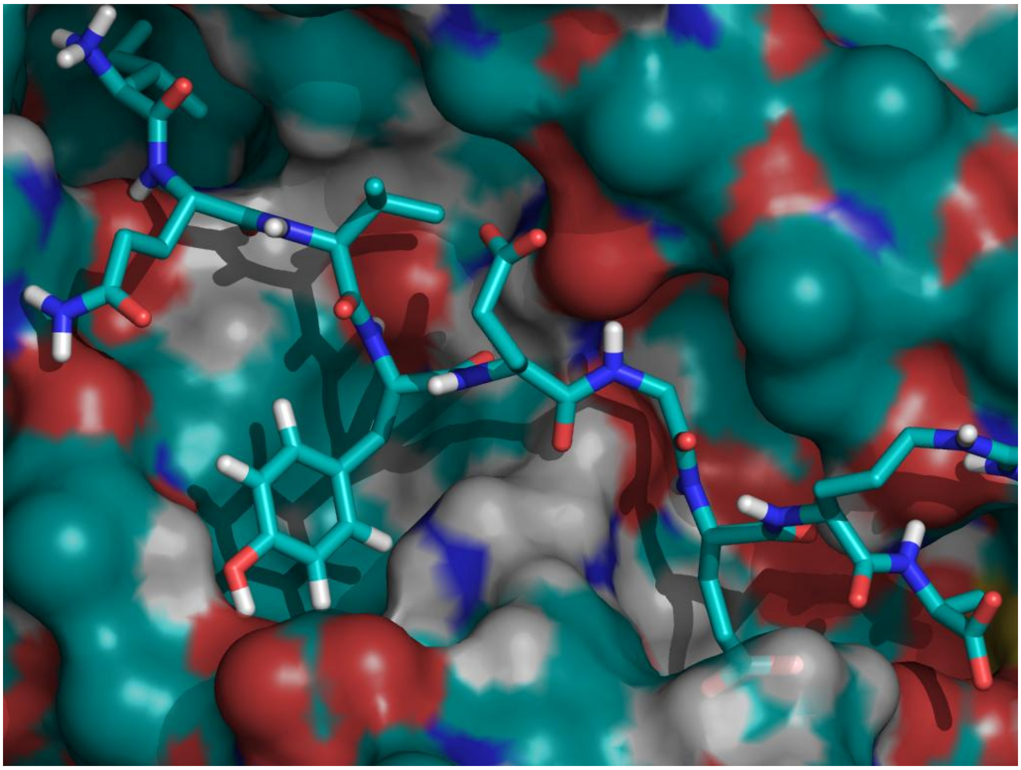

IQVYEGERA DR2b

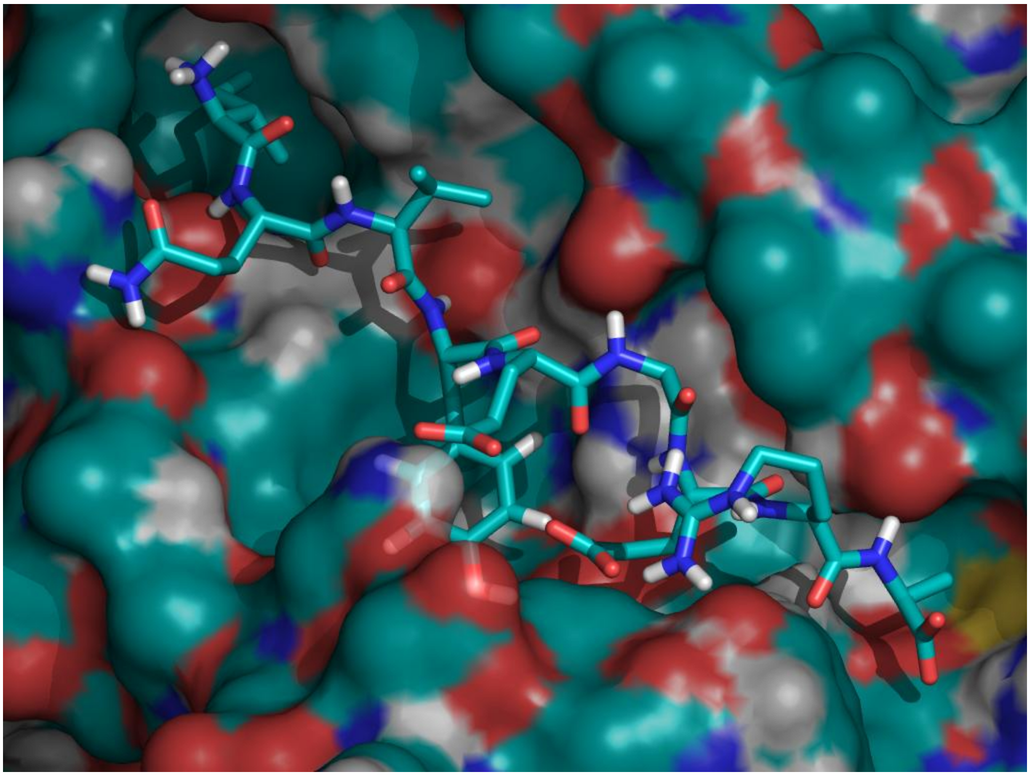

PKIQVYSRH DR2a

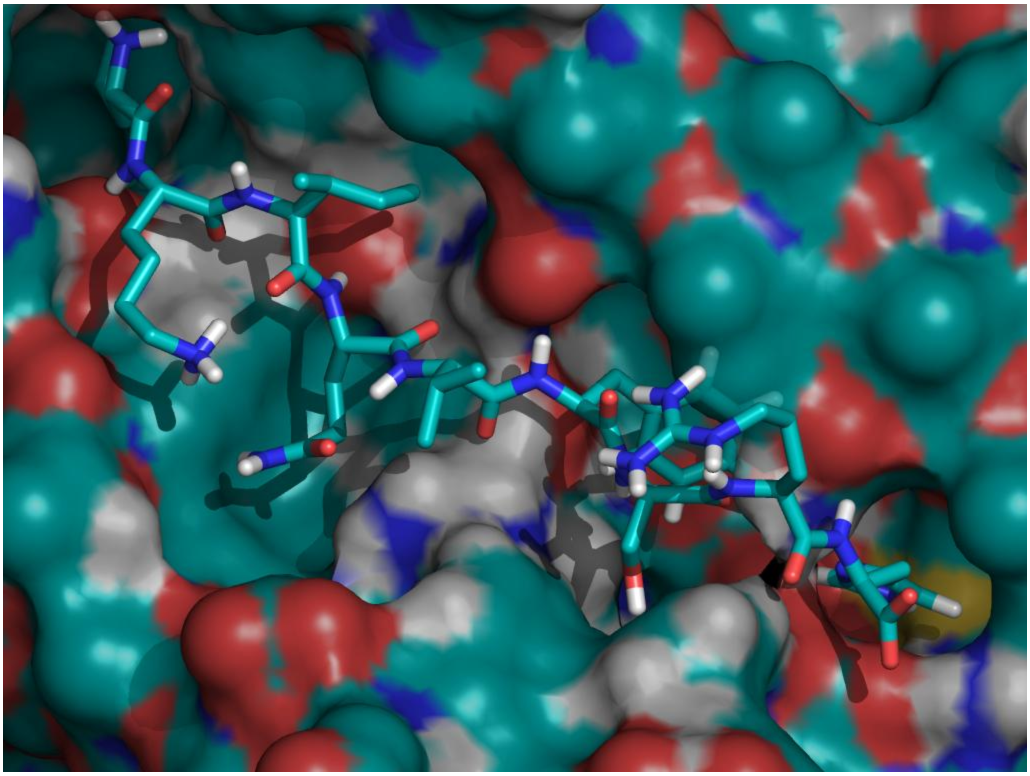

PKIQVYSRH DR2b

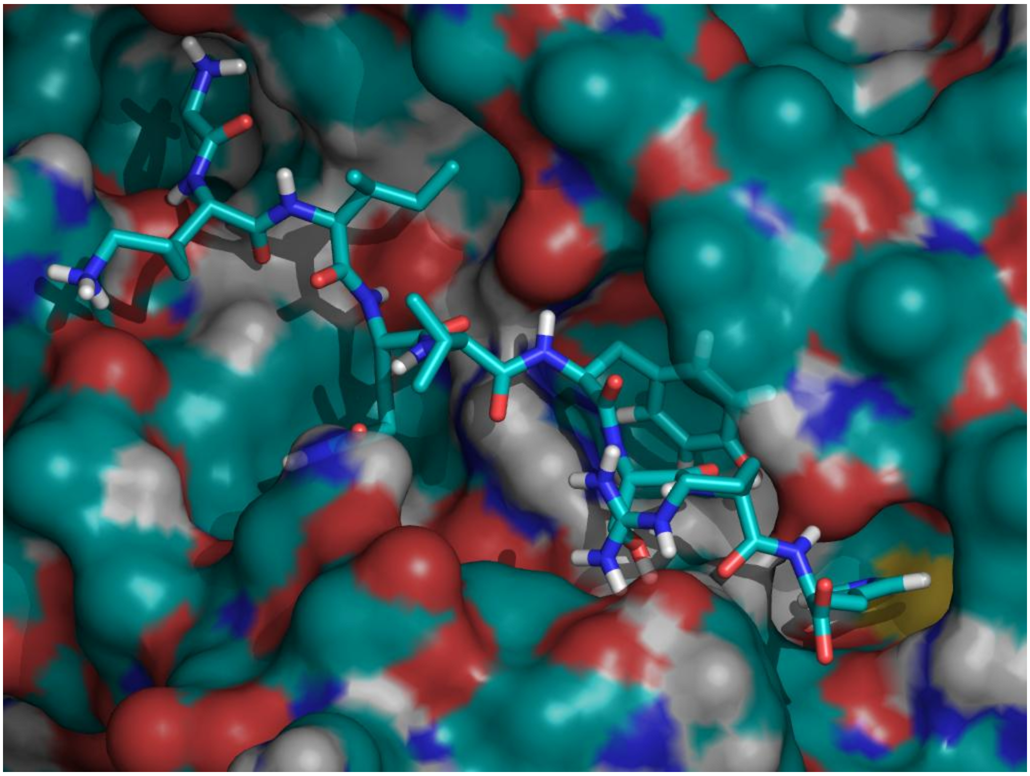

IQTERAYQK DR2a

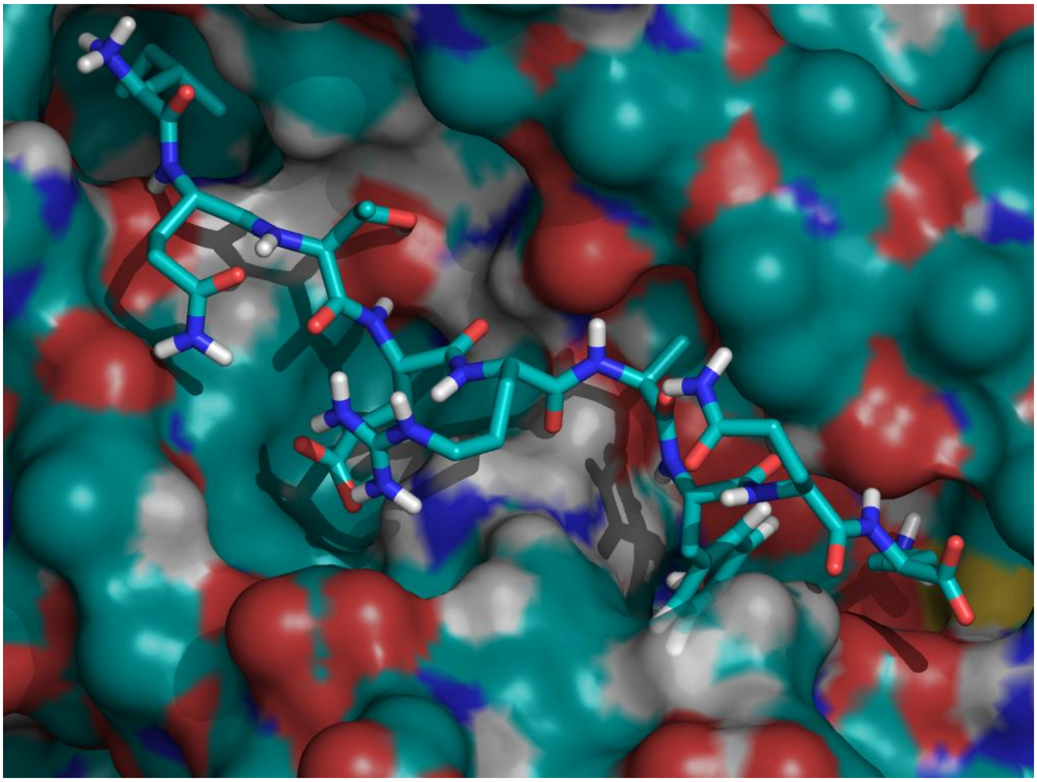

IQTERAYQK DR2b

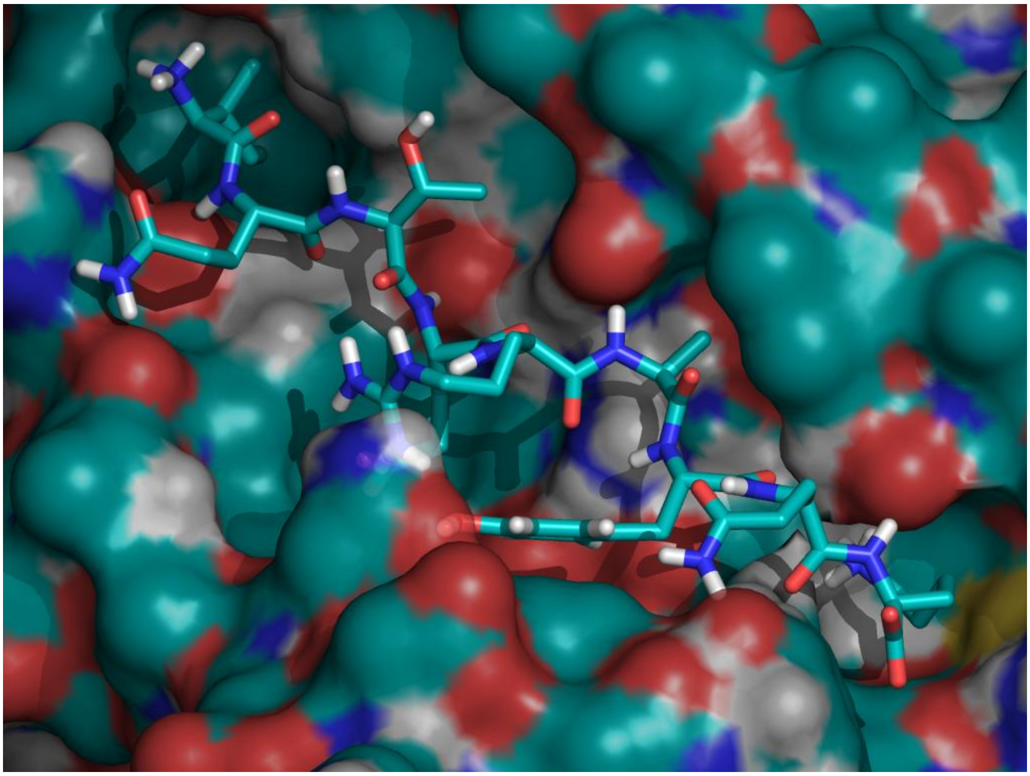

LRAEQQRLK DR2a

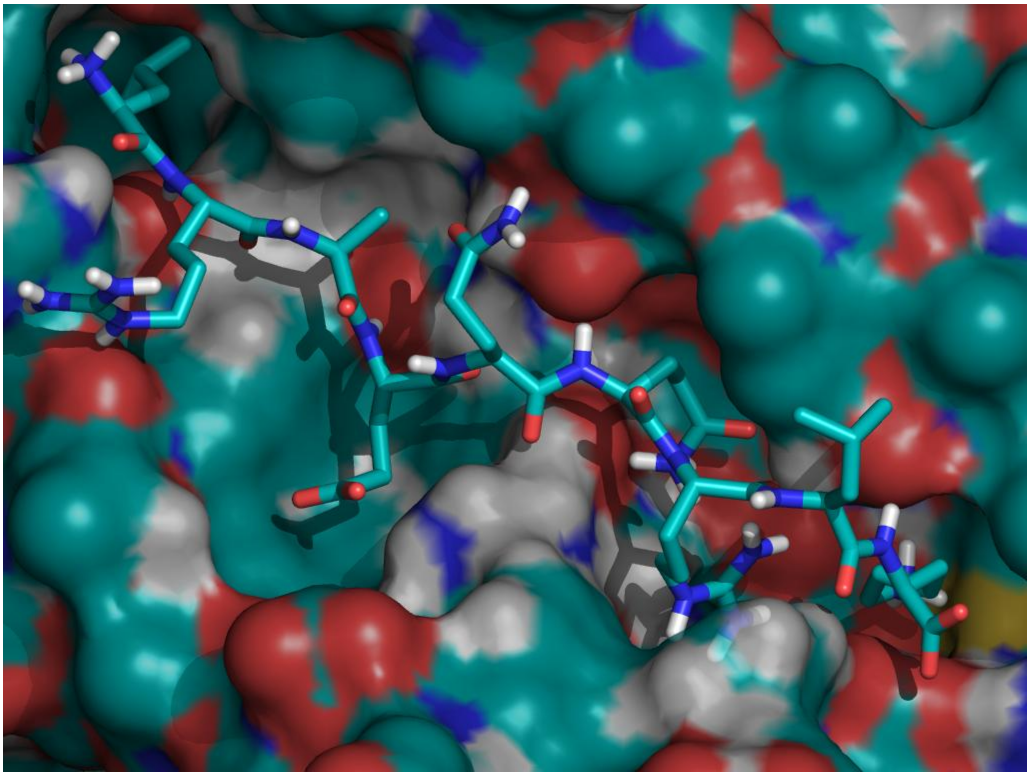

LRAEQQRLK DR2b

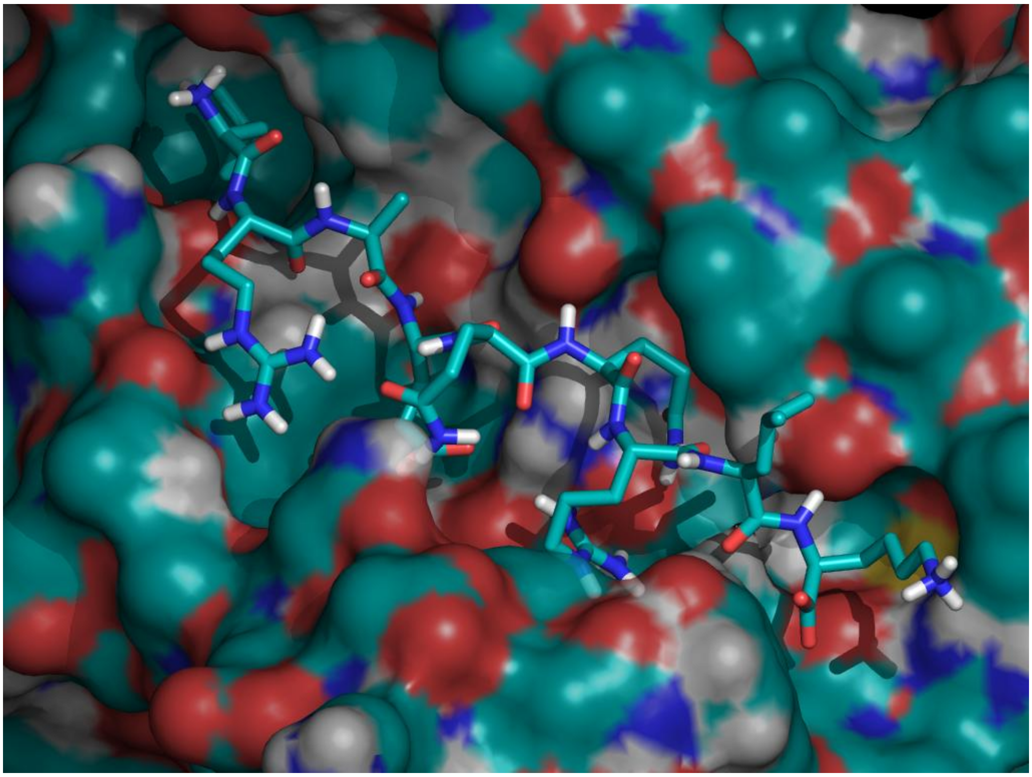

IVVQPGHIR DR2a

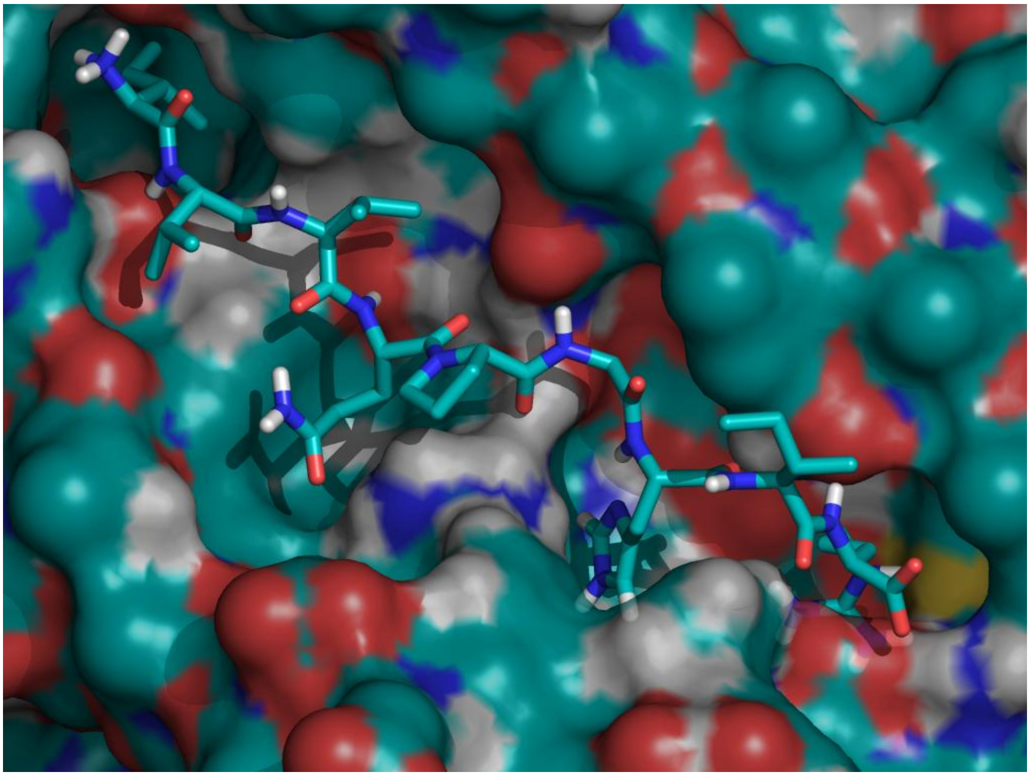

IVVQPGHIR DR2b

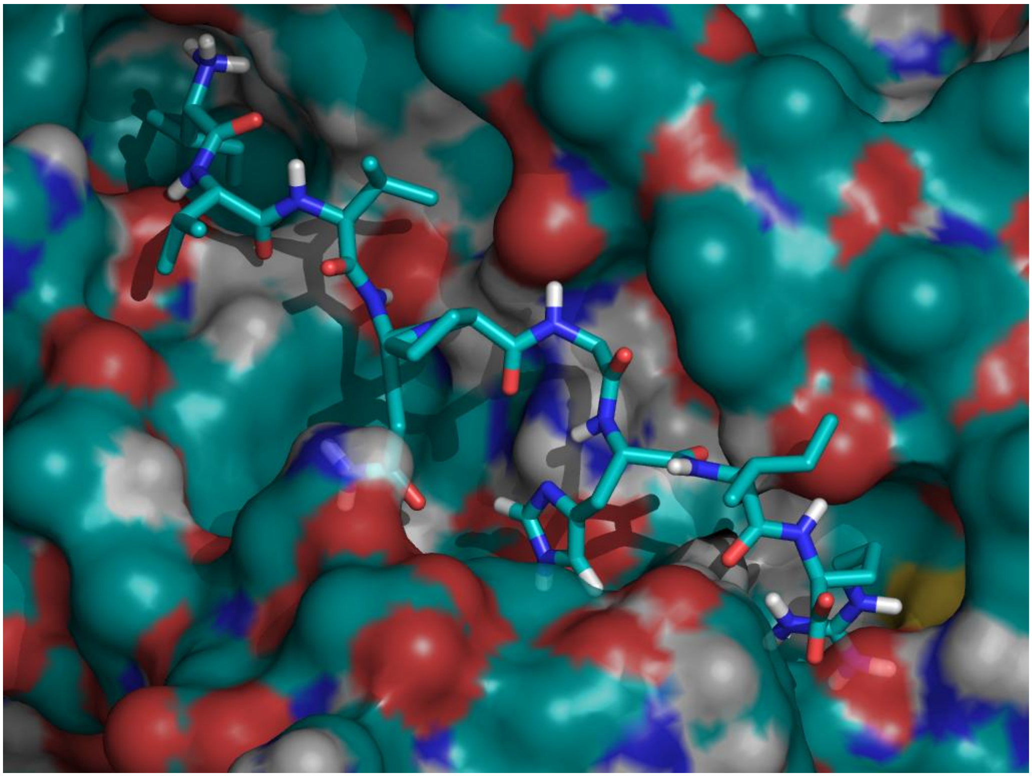

FQVLKSLGK DR2a

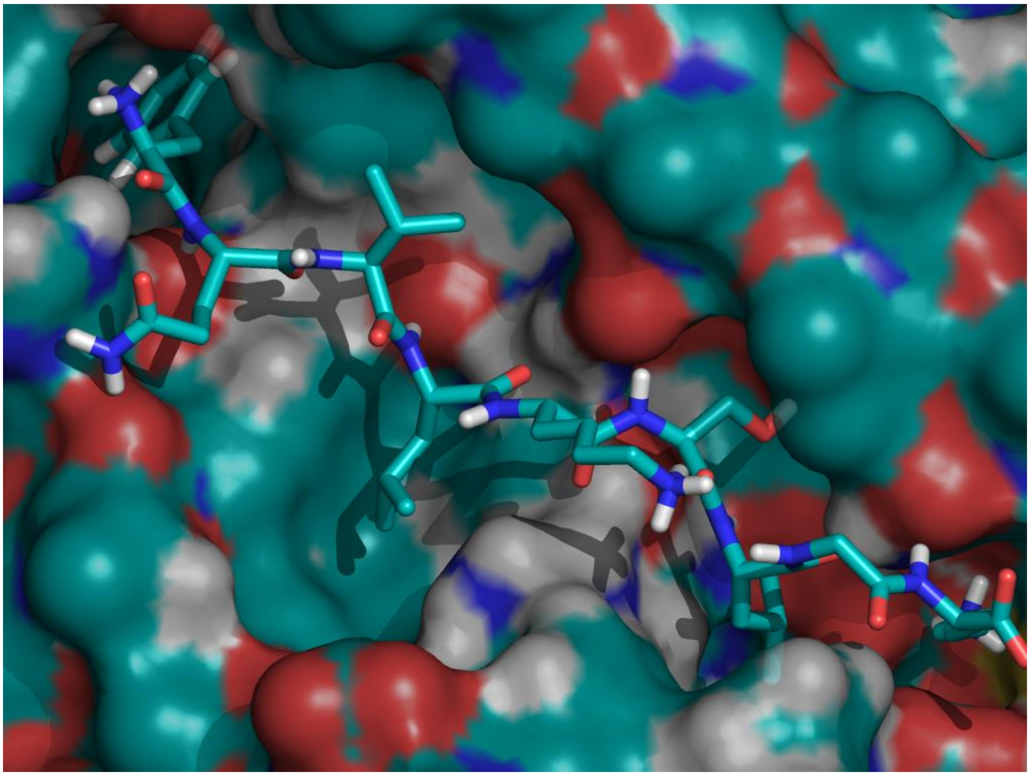

FQVLKSLGK DR2b

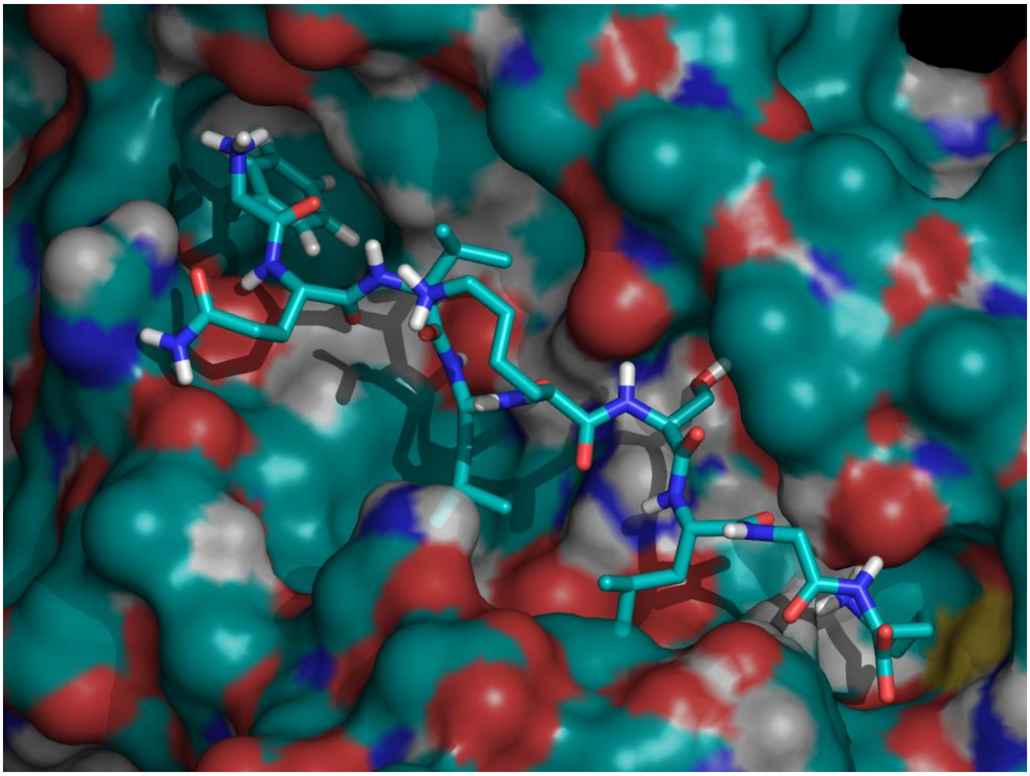

IVQAVSAHR DR2a

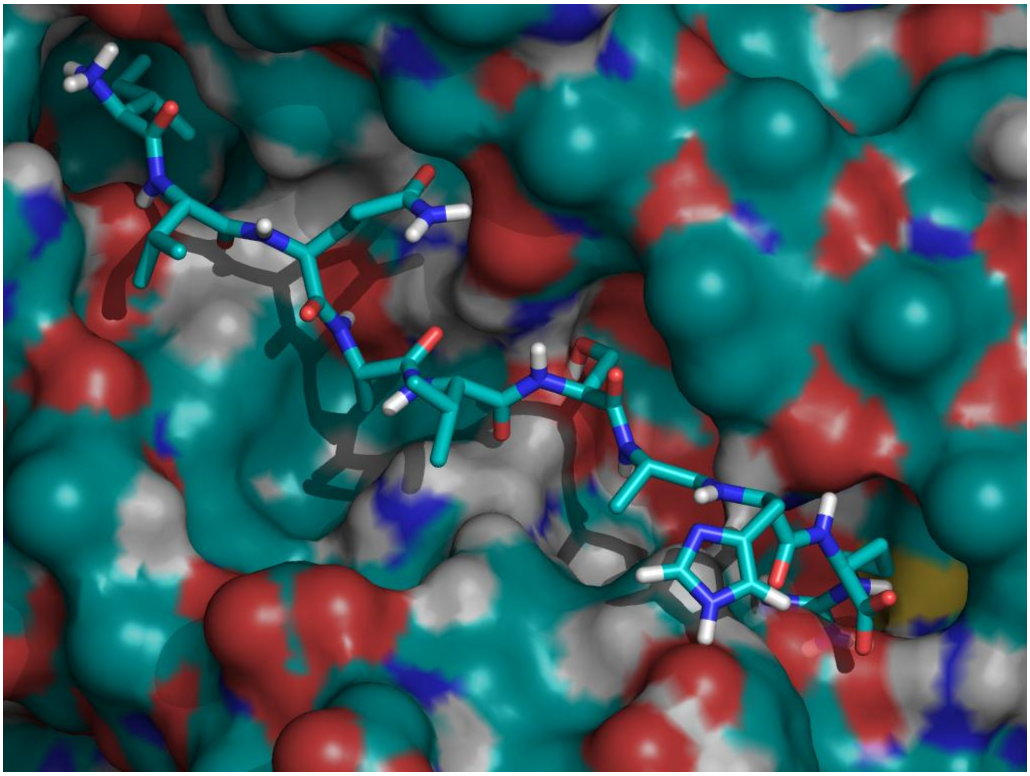

IVQAVSAHR DR2b

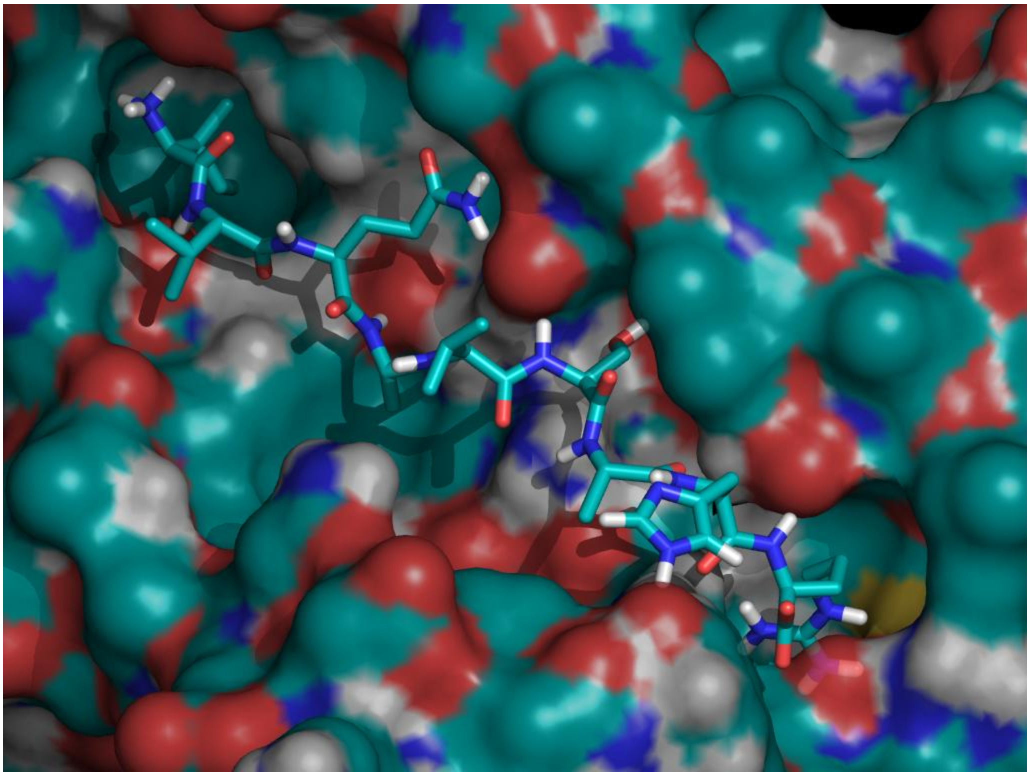

Supplement: Figure S3 — Model structures of DR15 ligands complexed with DR2a and DR2b. The interactions of the identified binding cores from peptides sequenced from MGAR (DR15) and from BLS-DR2a or -DR2b and DR2a and DR2b were modeled. Figures show the graphical representation of the models most favorable energetically. [file Image_3.PDF]
